# Retrospective descriptive assessment of clinical decision support medication-related alerts in two Saudi Arabian hospitals

**DOI:** 10.1186/s12911-022-01838-1

**Published:** 2022-04-15

**Authors:** Jamilah Ahmed Alsaidan, Jane Portlock, Sondus I. Ata, Hisham S. Aljadhey, Bryony Dean Franklin

**Affiliations:** 1 King Saud University, Riyadh, Kingdom of Saudi Arabia; 2 School of Life Sciences, University of Sussex, Brighton, UK; 3 Department of Pharmacy Services, King Saud University Medical City, Riyadh, Saudi Arabia; 4 Saudi Food and Drug Authority, Riyadh, Saudi Arabia; 5 UCL School of Pharmacy, University College London, London, UK

**Keywords:** Decision support systems, Clinical (MeSH), Computerised provider order systems (MeSH), Medication error (MeSH), Medication alert systems (MeSH)

## Abstract

**Objectives:**

To determine the frequency of clinical decision support system (CDSS) medication-related alerts generated, accepted, or overridden, to assess appropriateness of alert display and overrides, and to characterise the documentation of clinician justification for these overrides in an academic medical centre in Saudi Arabia.

**Materials and methods:**

System-generated CDSS reports for the period June 2015 to December 2017 were retrospectively reviewed and analysed. Alerts were classified into different types, and rates of alert overrides calculated as percentages of all generated alerts. A subset of 307 overridden alerts was assessed for appropriateness of display and override by two clinical pharmacists. Physician documentation of reasons for overriding alerts were categorised.

**Results:**

A total of 4,446,730 medication-related alerts were generated from both inpatient and outpatient settings, and 4,231,743 (95.2%) were overridden. The most common alert type was ‘duplicate drug’, accounting for 3,549,736 (79.8%) of alerts. Of 307 alerts assessed for appropriateness, 246 (80%) were judged to be appropriately displayed and 244 (79%) were overridden appropriately. New drug allergy and drug allergy alerts had the highest percentage of being judged as inappropriately overridden. For 1,594,313 alerts (37.7%), ‘no overridden reason selected’ was chosen from the drop-down menu.

**Conclusions:**

The alert generation and override rate were higher than reported previously in the literature. The small sample size of 307 alerts assessed for appropriateness of alert display and override is a potential limitation. Revision of the CDSS rules for alerts (focusing on specificity and relevance for the local context) is now recommended. Future research should prospectively assess providers’ perspectives, and determine patient harm associated with overridden alerts.

**Supplementary Information:**

The online version contains supplementary material available at 10.1186/s12911-022-01838-1.

## Introduction

Computerised provider order entry (CPOE) enables health care providers to enter orders for procedures and medication directly into a computer [1] rather than on paper [2]. CPOE ensures legible, complete orders [3], and allows incorporation of clinical decision support systems (CDSS) [1]. CPOE and CDSS have been adopted for the prescribing of medication in developed as well as developing countries, in both inpatient and outpatient settings. Despite widespread CPOE and CDSS adoption in hospitals around the world, the quality of evidence about their effectiveness in medication error and associated harm reduction is variable [4]. There are also differences in practising health care personnel, patient demographics, and delivery of quality of care, and thus transferability of the results of these evaluations to different settings cannot be assumed [5]. CDSS medication-related alert generation has been measured and alert override rates have been characterised in many developed countries [5–9] and in the region of the World Health Organization Eastern Mediterranean Regional office [10, 11] but studies from Saudi Arabia and other developing countries were limited. An overload of medication-related alerts could result in increased risk of alert fatigue, important alerts being ignored along with unimportant ones, and a false sense of security with an increased risk of adverse events [12]. From studies in the Kingdom of Saudi Arabia, one study found a significant difference (44.8% vs 35.8%) in drug related problem incidence pre- and post-CPOE implementation (p < 0.01). The authors concluded that the CPOE system significantly reduced drug related problems in this paediatric population [13]. Another Saudi Arabian study found no significant difference in the incidence of medication errors detected between the period of activation of one type of CDSS alert and a period of deactivation [14]. However, the types and numbers of alerts generated and overridden in developing countries are not well characterised. The aim of this study was to evaluate utilisation of CPOE and CDSS in King Saud University Medical City (KSUMC) hospitals and to develop generalisable recommendations for improved medication-related alert designs and alert handling practises. Our objectives were to determine numbers of different types of medication-related CDSS alerts generated, accepted and overridden, to assess the difference in the rates of alert override over time, to characterise the documentation of justification for these overrides, and to assess the appropriateness of alert display and overrides.

## Materials and methods

### Setting

The study took place in KSUMC, a tertiary care academic medical city in Riyadh, Kingdom of Saudi Arabia. KSUMC comprises two hospitals: King Khalid University Hospital (850 inpatient beds) and King Abdulaziz University Hospital (250 beds). KSUMC introduced CPOE for all physician orders including medication orders (Cerner® Millennium Version 2014) in May 2015, after which use of handwritten medication prescriptions ceased. CDSS alerts are produced by linking patient-specific information in the electronic health record and pharmacy records (the patient’s active current medication list, and their past medication list) with evidence-based knowledge to generate case-specific messages through rule-based software. The rules were partly based on the standard knowledge base provided as part of the CPOE system, and partly tailored by clinical pharmacists to be in concordance with local practice. At the time of this study, eleven types of medication-related alerts were enabled. Seven of these alert types: dose range, egg allergy, vincristine dose, switch medication from injectable to oral form, lack of documentation of weight and height, stop order, and glucose-6-phosphate dehydrogenase deficiency syndrome (G6PDD syndrome) incorporated local tailoring. Dose range alerts are generated when for example a physician needs to prescribe nine milligrams of warfarin and selects different available tablet strengths from the drop-down menu to complete the nine milligrams required; several drug duplicate alerts could be generated in the process. Further details on the alert types are available in the supplementary material, Additional file 1: Appendix A. All alerts were set up as ‘soft-stops’, providing information to the clinician [15], rather than as ‘hard-stops’ that prevent the clinician from proceeding. When an alert appears, the ordering physician therefore had the option of cancelling the prescribed medication, modifying it, or overriding the alert without making any changes. If the alert was overridden, the physician was required to select a reason for this from among ten pull-down reasons; after which a prompt appeared for entry of reason for override as free text.

### Design

This was a cross-sectional, retrospective, observational study of alerts generated by the CDSS system.

### Data sources

We used system-produced reports of medication orders and medication-related CDSS alerts for both inpatients and outpatients, including documented reasons for alert overrides. Monthly data were obtained for the period June 2015 to December 2017 and exported into Microsoft® Excel 2013. The patient’s electronic health record was accessed to assess the appropriateness of both the alert display and any overrides, as well as for characterisation of physician documentation for reason of override.

### Ethical approval

The study was classified as a service evaluation by University College London (UCL) School of Pharmacy, with UCL data protection registration obtained (reference Z6364106/2017/04/04). The study protocol was approved by King Saud University Institutional Review Board (reference 17/0431/IRB).

### Piloting

A pilot study of 100 randomly selected alerts generated from the inpatient and outpatient setting (50 from each setting) was first conducted for three purposes. There were three purposes for the pilot study. The first was to inform a decision on the sample size [16] for the main study. The second purpose was to develop the criteria for assessment of alert display appropriateness, and alert override appropriateness. The third was to calculate inter-rater agreement [17] on the alerts’ assessment of appropriateness of overrides and the appropriateness of alert display. The percent assessed to be overridden appropriately was then used to calculate a manageable sample size that would result in a suitable 95% confidence interval around the actual proportion of overridden alerts. The 100 orders assessed were selected by use of a random number generator [18].

### Data analysis

#### Alert data analysis

**Fig. 1:**
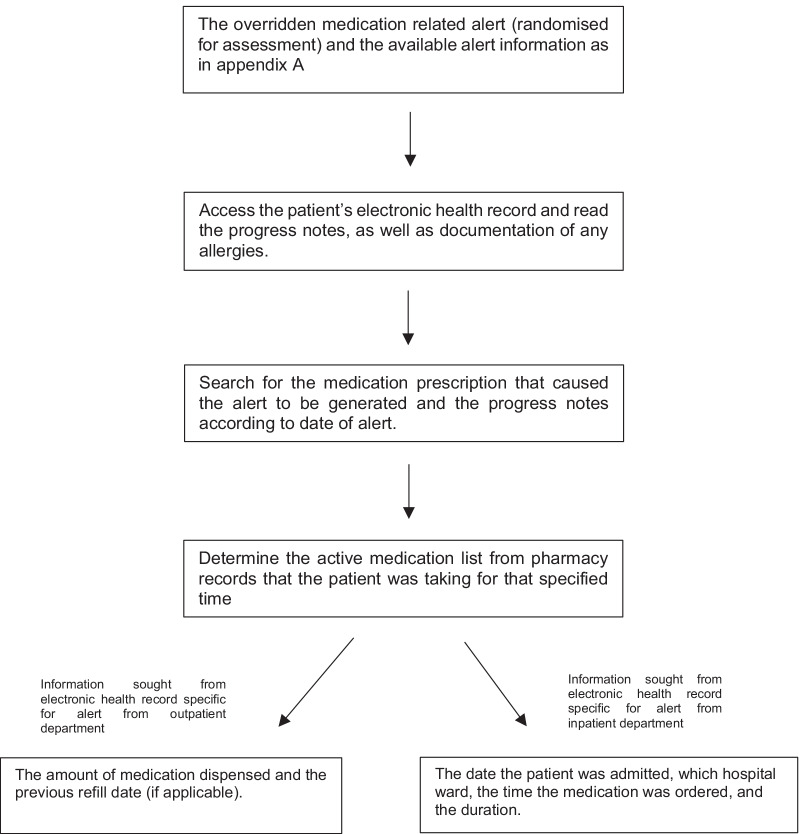
General process of seeking information from the electronic health record during assessment of alert display and assessment of alert override

After the pilot, the calculated random samples of overridden alerts were assessed independently by two researchers (JA and SA); their individual judgements were then compared, and any differences discussed until consensus was reached. If consensus could not be reached, discrepancies were referred to a third assessor, a practising clinical pharmacist at KSUMC. Criteria to assess appropriateness of alerts display and alert overrides were developed through review of previous studies [7, 8, 11, 19–21], and adapted through discussion among the research team to reflect local practice at KSUMC. The criteria developed and the parameters assessed for each alert type to determine the appropriateness of alert display and appropriateness of alert handling can be found in Additional file 1: Appendix B. During assessment of alert display the rules of generation of the alert type were considered; if it was displayed in concordance with the rules of its generation it was assessed as appropriately displayed. Alert overrides were assessed taking into consideration the clinical scenario of the patient; this information was sought from the electronic health record. Even if an alert was displayed in concordance with the rules of its generation and thus considered appropriate, according to an individual clinical scenario the physician’s decision to override the alert and prescribe the medication for the patient could be considered as appropriate. Figure 1 summarises the general process of seeking patient-related information from the electronic health record and pharmacy records during assessment of appropriateness of alert display and alert override. Details of the processes followed during assessment can be found in Additional file 1: Appendix C, with examples of assessed alerts in Additional file 1: Appendices D and E. The reasons for alert overrides as documented by physicians were classified into mutually exclusive categories. The categories were developed by repeatedly reading through and becoming familiar with clinicians’ documented free text reasons and review of previous studies [11, 19–21] and adapted through discussion among the research team**.** All patient and physician identifiers were removed to protect identities.

### Statistical analysis

Microsoft® Excel (2013, 2016) was used for the statistical analysis. Alert generation and override data were reported as counts and percentages. Inter-rater agreement was calculated as Cohen’s Kappa. The Chi-squared test was used to assess the difference in the rates of alert overridden across the three different calendar years. A p-value of less than 0.05 was specified a priori as being statistically significant. The Strengthening the Reporting of Observational Studies in Epidemiology (STROBE) recommendations were used to guide the reporting of this study (Additional file 1: Appendix O).

## Results

### Characterisation of alert generation and override

Between June 2015 and December 2017, a total of 5,114,310 medications were ordered, leading to the generation of 4,446,730 alerts, of which 4,231,743 (95.2%) were overridden. Within the outpatient department, 4,251,481 medications were ordered and 1,652,434 alerts generated of which 1,616,437 (97.8%) were overridden. For inpatients, 862,829 medications were ordered and 2,794,296 alerts generated, of which 2,615,306 (93.6%) were overridden. The most common type of alert, accounting for almost 80% of alerts, was ‘drug duplication’, followed by ‘dose range’ and ‘drug interaction’ alerts (Table 1 and Additional file 1: Appendices F and G). Medications most commonly triggering dose range alerts were potassium chloride, insulin, heparin, warfarin and pantoprazole. Drug allergy alerts made up less than 1.0% (n = 12,187), with 11,122 (91.3%) overridden.

**Table 1:** The number and percentage of alerts generated and overridden per alert type during the study period

Alert type	Generated		Overridden	
	Number	Percentage of total generated alerts (%)	Number	Percentage of alerts overridden
Switch medication from injectable form to oral	23,345	0.5	22,839	97.8
Dose range	491,830	11.1	480,898	97.8
Drug interaction	318,809	7.2	310,096	97.3
Drug duplicate	3,549,736	79.8	3,362,733	94.7
Vincristine	17	0.000	16	94.1
Drug allergy	12,187	0.3	11,122	91.3
Documentation of height and weight	47,927	1.1	43,089	89.9
G6PD-syndrome	82	0.00018	66	80.5
Egg allergy	226	0.005	145	64.2
New drug allergy	1455	0.033	718	49.3
Stop order	1116	0.0251	21	1.9
Total	4,446,730	100	4,231,743	95.2

The alert type ‘switch medication from injectable form to oral was deactivated after May 2016

Of 226 egg allergy alerts generated, 145 (64.2%) were overridden, mostly for vaccines in the paediatric population and emulsions prescribed as part of parenteral nutrition. The rates of alert override differed significantly across the years of the study for all alert types (*p* < 0.000), except for the alert type “vincristine” (*p* = 0.223). While differences were statistically significant because of the large sample size; the actual percent differences were generally small (Additional file 1: Appendix H).

The medication alert relating to switching medication from the injectable to the oral route was deactivated after May 2016, (this alert was switched off after being active for less than one year), the decision was unrelated to the study. Thus, the difference in rates of alert override over the years 2015, 2016, and 2017 could not be calculated for this alert.

### Reasons for overrides

Table 2 shows the number of times each reason was selected from the (eleven option) drop-down menu for both outpatient and inpatient settings. For 1,594,313 (37.7%) of overridden alerts, no reason was selected. The most commonly selected reason from the pull-down menu chosen for 1,835,154 (43.3%) of overridden alerts was ‘physician approved override’ followed by ‘physician reviewed drug interaction’ chosen for 329,606 (7.8%) overridden alerts. Free text was entered for 13,461 (less than 0.5%) of overrides, of which 9158 entries (68%) were non-sensical characters such as one letter only (‘S’) or one letter repeated (‘CCCC’) or several letters that do not spell a word (such as ‘Gjgfh’). The remaining 4,376 free text entries (32%) were designated into categories as outlined in Additional file 1: Appendix I with actual examples of entries. The categories developed were broadly either special instructions or patient-centred factors to justify overriding the medication related alert display. Examples of categories of special instructions were ‘As instructed by physician or consulted with other health care practitioners’ and ‘As per protocol or policy’. An example of a patient-centred factor was ‘patient special circumstances’. Some examples of verbatim entries are ‘as order by consultant dr.’, ‘DR DECISOON’ [sic], ‘allowed by doctor’ and ‘Other DKA [diabetic ketoacidosis] protocol’, ‘pt [patient] is struggeling [sic] with refills that are not synchronised [sic]. Thus pt [patient] was given all prescriptions today’.

**Table 2:** Reasons selected upon overriding alert from pull-down menu or entered as free text

Reasons from drop down menu	Total	Percentage of total reasons this option was selected (%)
Physician Approved Override	1,835,154	43.366
No Overridden Reason Selected	1,594,313	37.675
Physician Reviewed Drug Interaction	329,606	7.789
Physician Clinical Judgement	231,177	5.463
Physician Addressed	125,788	2.972
Physician Previously Reviewed	32,648	0.772
Other	32,141	0.760
Pharmacist Clinical Judgement	19,077	0.451
Consulted Physician, OK Received	14,389	0.340
Free text entries	13,461	0.318
Pharmacist Reviewed Drug Interaction	2,061	0.049
Pharmacist Reviewed Allergy	1,928	0.046
Total	4,231,743	100

### Results of pilot study

Of the 100 randomly selected alerts for the pilot study, the electronic health record could not be accessed for eight alerts thus 92 alerts were assessed**.** Fifty-four of the 92 alerts (59%) were assessed to be appropriately overridden in the pilot study. Therefore, around 0.59 were expected to be appropriate overall. This proportion was used to calculate that a sample size 307 alerts (154 alerts from inpatient setting, 153 alerts from the outpatient setting) would result in a 95% confidence interval of 0.53 to 0.64. The measure of agreement between the two assessors on the decisions regarding the judgment of alert display appropriateness and appropriateness of alert override, Cohen’s Kappa was calculated to be 0.62 and 0.71, respectively. This was interpreted as substantial agreement [17]. There were no instances where consensus between the two researchers could not be reached through discussion, and referral to a third evaluator was not necessary. The following demographic data are for the subset of 307 alerts assessed for appropriateness of display and override.

### Results of assessment of subset of alerts for appropriateness

#### Demographic data of inpatients

As for the demographic data of the patients for which the medications were ordered from the inpatient department, the median age of the patients with these 154 documented alerts was 33.3 years (interquartile range (IQR) 6.8–58.0) across 52 different nursing units in King Khalid and King Abdulaziz University Hospitals. Eighty-three of the 153 (54%) sampled alerts were for medications prescribed for females, and 71 (46%) for males.

#### Demographic data of outpatients

From the outpatient department (from 34 outpatient clinics in both hospitals of KSUMC) the median age of the patients who had these 153 documented alerts was 35.5 years (IQR = 27.5–55.3). Of the 153 alerts assessed, seventy-one (46%) of the sample were females, and 82 (54%) were males.

#### Assessment of alert display

Regarding alert display, of the total of 307 alerts assessed, 246 (80.1%) were assessed as having been displayed appropriately. Of the 37 drug duplicate alerts assessed, 32 (86.5%) were assessed to be displayed appropriately. Assessed alerts from the alert types’switch medication from injectable dosage form to oral’ and ‘vincristine dose’ had the lowest percentage of display appropriateness (Table 3, and Additional file 1: Appendices J and K).

**Table 3:** The percentage and number of alerts assessed to be displayed appropriately for each alert type from the inpatient and outpatient setting

Alert type	Number of alerts (outpatient settings)	Number of alerts (inpatient settings)	Total number of alerts	Number of alerts displayed appropriately	Percentage of alerts appropriately displayed
Egg allergy	22	18	40	40	100.0
Glucose 6 Phosphate dehydrogenase deficiency syndrome	0	16	16	16	100.0
Drug interaction	22	15	37	35	94.6
Drug allergy	21	16	37	32	86.5
New drug allergy	21	16	37	32	86.5
Drug duplicate	21	16	37	28	75.7
Documentation of height and weight	22	16	38	25	65.7
Dose range	22	14	36	22	61.1
Switch medication from injectable form to oral	2	16	18	11	61.1
Vincristine	0	11	11	5	45.5
Total	153	154	307	246	80.1

Regarding assessment of alert overrides, of 307 alerts assessed 244 (79.5%) were assessed to be overridden appropriately. All assessed alerts of the type ‘switching medication from injectable form to oral’ were assessed to be overridden appropriately. Of eleven assessed overridden vincristine dose alerts, seven (63.3%) were assessed to be overridden appropriately. The assessed alerts of types ‘drug allergy and ‘new drug allergy’ alert type had the lowest percentage of override appropriateness (Table 4, Additional file 1: Appendices L and M).

**Table 4:** The percentage and number of alerts assessed to be overridden appropriately for each alert type from the inpatient and outpatient according to setting

Alert type	Alerts assessed from the outpatient setting	Alerts assessed from the inpatient setting	Total number of alerts assessed	Total number of overrides considered appropriate	Percentage of alerts overridden appropriately
Switch medication from injectable form to oral	2	16	18	18	100
Egg allergy	22	18	40	38	95.0
Glucose 6 Phosphate dehydrogenase deficiency syndrome	0	16	16	15	93.8
Dose range	22	14	36	32	88.9
Drug duplicate	21	16	37	32	86.5
Documentation of height and weight	22	16	38	32	84.2
Drug interaction	22	15	37	31	83.9
Vincristine	0	11	11	7	63.6
Drug allergy	21	16	37	23	62.2
New drug allergy	21	16	37	16	43.2
Total	153	154	307	244	79.5

## Discussion

### Summary of key findings

In this study, after a retrospective report review, it was found that a total of 5,114,310 medications were prescribed and led to generation of 4,446,730 alerts. From the outpatient department, 38 alerts were generated per 100 medications ordered, while from the inpatient, 323 alerts were generated per 100 medications ordered. Ninety five percent of all alerts were overridden, and for 37.7% no reason for the override was documented or chosen from the menu. Of the 307 alerts assessed for appropriateness, 40 (100%) of assessed egg allergy alerts and 16 (100%) of G6PDD syndrome alerts were displayed appropriately, while new drug allergy and drug allergy alerts were most commonly judged inappropriately overridden.

### Interpretation of findings

In our study, the alert generation rate and override rate were considerably high for all alert types, and it was found that alert types most commonly judged to be overridden inappropriately were new drug allergy and drug allergy alerts, potentially exposing the patient to adverse events ranging from hives to anaphylactic shock. Some of our results are similar to existing international studies; for example drug duplicates were reported to be the most commonly generated alert type in previous studies in outpatients in the United Kingdom [6] and the United States of America [7], and were the type most likely to be overridden [6]. The very high frequency of the display of drug duplicate alerts from both inpatient and outpatient settings at KSUMC could be a cause of alert fatigue for the physicians, as found by earlier studies [22]. In an inpatient study in the United States of America, about 73% of patient allergy, drug interaction and duplicate alerts were overridden, with 60% of the overrides judged to be appropriate [9]. Some of our findings are also different to existing studies. For example, the alert generation rate per 100 medications ordered was higher than reported previously by a study in the United Kingdom, where 13 medication related alerts were visible to the user per 100 medications ordered [6]. In a study on computerised drug alerts in primary care in the United States of America [23] it was judged that 69 (36.5%) of alerts were inappropriately displayed or invalid, which was higher than in our study which found only 19.1% of alerts to be displayed inappropriately. There is also a potential difference in sample characteristics as the median age of inpatients in our study was only 33.3 years, most likely due to the relatively young population of Saudi Arabia [24].

This study is the first study in Saudi Arabia to report rates of generation of G6PDD syndrome alerts and override rates, and assess the appropriateness of display and override. The use of this alert type was not reported in previous studies. This study also adds to the literature reporting poor documentation of reasons for overriding medication related alerts with more than half of free text entries being non-sensical characters without proper reasoning.

### Recommendations for practice

Increased understanding of the processes (of alert generation and alert override) can lead to the development of recommendations that possibly to lead to the refinement of the generated and displayed alerts and have potential to reduce generation and display of clinically irrelevant alerts, and thus potentially reduce experienced alert fatigue. The main recommendation is to revise the rules responsible for alert generation [25]. An example would be to suppress generation of alerts from a patient’s past inactive medication list, which currently leads to irrelevant drug duplicate and drug interaction alert generation. Another suggestion would involve revision of the dose ranges and thresholds currently activated for the dose range alert and vincristine alert. These two alert types had high override rates despite previous tailoring for KSUMC practice. The third suggestion would be to introduce ‘a level of interaction’ or tiered alerts determining generation and display of drug interaction alert, as it could potentially reduce irrelevant drug interaction alerts.

The second recommendation would be to introduce a ‘hard stop’ as an action after the display of an alert, ensuring the physician cannot proceed with the order of this medication. It would be particularly useful for example for drug interaction alerts with risk level D or X, and for drug allergy alerts of level D or X, and for egg allergy alerts (Additional file 1: Appendix N) according to online drug information reference available at KSUMC [26].

The third recommendation would be to removal of the option ‘no reason selected’ when overriding an alert, thus would remain ten options to choose from the drop-down menu in addition to the choice of entry of free text. The ten options appearing could be customised according to alert type. There is a potential benefit to be realised by using a customised list for medication override reasons [21]. It is suggested to educate practitioners on the importance of documentation of accurate reason for overriding an alert by continuous training sessions, online or practical sessions when feasible and incentives, such as guaranteed confidentiality [27] upon analysis of entries.

A final recommendation would be to assign a team including health care practitioners as part of a committee to review and implement CDSS changes. A study from the United Kingdom has recommended medication prescribers and other users be involved in design of CDSS alerts into prescribing workflows [28] which could also enhance targeting generation of alerts at the right time.

### Study strengths and limitations

Among the strengths are that alerts were assessed individually by two clinical pharmacists. Interrater agreement was calculated and interpreted as being substantial. Alert generation and override rates were calculated for all medications ordered from both inpatient and outpatient settings. Criteria used to assess appropriateness of alert display and appropriateness of alert override in this study were adapted from existing criteria [7, 8, 11, 19–21], with these criteria now available for future research.

This study had several limitations. The first is representativeness or generalisability, as it was conducted in one medical city using one CPOE system, and the 307 randomly selected alerts assessed for appropriateness may not be generalisable to the whole population. The second is that as the main source of patient-related information for assessment of appropriateness of alert display and override was the electronic health record; information not documented would not be taken into account [29]. The third limitation is that alert overrides and reasons for overriding alerts were not classified according to physician speciality to protect physician anonymity. The fourth limitation was that any resultant effect on patient outcomes (e.g., adverse events) were not investigated. Lastly, the alerts that physicians accepted (resulting in cancelation or modification of the medication order) could not be analysed further than their frequency as their details did not appear in the data reports accessed. Analysis of accepted medication-related alerts, their appropriateness and under which circumstances these alerts are accepted, could be studies in future to help understand what qualifies as a ‘successful and clinically relevant’ alert [30].

## Conclusions

The findings of this research have shed light on medication-related alert generation and handling in a university medical city after implementation. Discovery of such high alert override rates, and poor documentation of reasons for overriding the alerts have been important for consideration to develop recommendations to improve medication related generation and alert handling with potential to improve clinical practice. Finally, the results of this research are a baseline to which future studies worldwide might refer. Implementing CPOE and CDSS without comprehensive functionality and processes in place to ensure meaningful system use does not necessarily decrease medication errors [31]. Researchers have cautioned against using override rates as a means of assessing alert effectiveness. This is primarily because knowing the override rate does not tell the full story an alerts impact on prescribing behaviour [32]. Possible areas for future research work include studies conducted with prescribers, to explore their perceptions regarding the generated medication related alerts, and to document their suggestions on how to improve their experiences of using CPOE and CDSS. Future research could also focus on how to obtain meaningful information for analysing documented reasons as entries for overriding alerts to ensure efficient system use [33].

## Supplementary Information

**Additional file 1**. **Appendix A:** Alerts’ description and setting which they generated. **Appendix B:** Criteria and parameters for assessment of appropriateness of alert display and appropriateness of alert override. **Appendix C:** Steps of assessment of appropriateness of alert display and alert override for each alert type. **Appendix D:** Example of assessed alerts for appropriateness (from inpatient setting). **Appendix E:** Example of assessed alerts for appropriateness (from outpatient setting). **Appendix F:** Alerts generated and overridden from inpatient setting. **Appendix G:** Alerts generated and overridden from outpatient setting. **Appendix H:** The rates of alert override across years of study. **Appendix I:** Classification into categories the examples of free text entries of reasons for overriding alerts. **Appendix J:** The percentage and number of alerts assessed to be displayed appropriately for each alert type in the inpatient setting. **Appendix K:** The percentage and number of alerts assessed to be displayed appropriately for each alert type in the outpatient setting. **Appendix L:** The percentage and number of alerts overridden appropriately for each alert type in the inpatient setting. **Appendix M:** The percentage and number of alerts overridden appropriately for each alert type in the outpatient setting. **Appendix N:** Meaning of interaction and allergy categories. **Appendix O:** Strengthening the Reporting Observational Studies in Epidemiology Statement—Filled Checklist of items included in reporting of this study.

## Data Availability

The datasets generated or analysed during the current study are not publicly available due to King Saud University Medical City policies but are available upon reasonable request from corresponding authors and with permission of King Saud University Medical City.

## Abbreviations

CDSS: Clinical decision support system
CPOE: Computerised provider order entry
G6PDD syndrome: Glucose 6 phosphatase dehydrogenase deficiency syndrome
KSUMC: King Saud University Medical City
STROBE: Strengthening the Reporting of Observational Studies
UCL: University College London
