# Supplementary material for: Retrospective descriptive assessment of clinical decision support medication-related alerts in two Saudi Arabian hospitals
Source: BMC Med Inform Decis Mak. 2022 Apr 15;22:101. doi: 10.1186/s12911-022-01838-1 (PMC9012024; doi:10.1186/s12911-022-01838-1)
Supplement: Supplementary file 1 — Additional file 1. Appendix A: Alerts’ description and setting which they generated. Appendix B: Criteria and parameters for assessment of appropriateness of alert display and appropriateness of alert override. Appendix C: Steps of assessment of appropriateness of alert display and alert override for each alert type. Appendix D: Example of assessed alerts for appropriateness (from inpatient setting). Appendix E: Example of assessed alerts for appropriateness (from outpatient setting). Appendix F: Alerts generated and overridden from inpatient setting. Appendix G: Alerts generated and overridden from outpatient setting. Appendix H: The rates of alert override across years of study. Appendix I: Classification into categories the examples of free text entries of reasons for overriding alerts. Appendix J: The percentage and number of alerts assessed to be displayed appropriately for each alert type in the inpatient setting. Appendix K: The percentage and number of alerts assessed to be displayed appropriately for each alert type in the outpatient setting. Appendix L: The percentage and number of alerts overridden appropriately for each alert type in the inpatient setting. Appendix M: The percentage and number of alerts overridden appropriately for each alert type in the outpatient setting. Appendix N: Meaning of interaction and allergy categories. Appendix O: Strengthening the Reporting Observational Studies in Epidemiology Statement—Filled Checklist of items included in reporting of this study. [file 12911_2022_1838_MOESM1_ESM.docx]

## Additional file 1:

Appendix A: Alerts’ description and setting which they generated.

| **Alert name** | **Meaning and significance** | **Setting the alerts are generated** | **Comments** |
| --- | --- | --- | --- |
| Drug duplicate | This alert is displayed when the medication has been prescribed for this patient before. One issue is that even if the patient has received this medication years ago, it will trigger this alert if it is in the patient’s current or past medication list.  This alert will also be triggered if a physician wants to prescribe a medication for a patient that is in the same pharmacological and therapeutic class.  In the inpatient setting when a physician's order is renewed for a patient medication that is taken daily for a week, or through the whole duration of the stay, and the duplicate alert will appear every single time. | Inpatient and outpatient |  |
| Dose range alert | This the alert triggered for dose range checking, indicating that the dose the physician has prescribed should be revised and checked, as it has been linked with the patient’s electronic health record, considering the patient's laboratory test values, and individualised parameters.  The recommended doses for some medications, (not all medications) have been checked according to the guidelines of practice at KSUMC and for patient characteristics. | Inpatient and Outpatient | This alert is only activated for certain medications.  Since system launch May 2015, the alert was activated for 18 medications  In January 2017 this alert was activated for an additional 54 medications (the dosage range information was revised and by KSUMC pharmacy staff).  In March 2017 an additional thirteen medications were activated for dose range checks, but without revision of information by clinical pharmacists at KSUMC.  There is a total of eighty-five medications that have been activated for dose range checking. |
| Drug interaction | The alert is displayed when there is a drug interaction.  Therefore, the alert appears to alert the prescriber when one medication is prescribed that interacts with a medication that was on the patient’s active and/or inactive medication list. | Inpatient and outpatient |  |
| Drug allergy | The alert is generated if there’s an existing allergy, and a new medication is prescribed to which the patient is allergic, or a possibility of having a reaction (for example the same pharmacological class). | Inpatient and outpatient |  |
| New drug allergy | The alert is generated if the medication has already been ordered and a new allergy to a medication is added to the information on the patient’s the electronic health record. | Inpatient and outpatient |  |
| Documentation of height and weight | If height and weight are missing for age<12 years, this alert will be displayed. | Inpatient and outpatient |  |
| Egg allergy | This alert indicates that the prescribed drug for this patient contains egg or egg protein derivative, and according to the information on in the electronic health record the patient is allergic to eggs/egg containing compound. | Inpatient and outpatient |  |
| Glucose-6-phosphate dehydrogenase deficiency syndrome | This alert indicates that the prescribed drug for this patient is not suitable according to the condition documented in the electronic medical record. This condition is Glucose-6-phosphate dehydrogenase deficiency syndrome, an inborn error of metabolism that predisposes to red blood cell breakdown. | Inpatient and outpatient |  |
| Switch medication from injectable form to oral | This alert indicates that the prescribed drug for this patient should be prescribed in the oral dosage form instead of injectable route of administration. | Inpatient | It appeared from May 2015 and last appeared 30 April 2016. As of 1 May 2016, this alert ceased to be generated, it was turned off officially. The reason is because of King Saud University Medical City policy change, which dictates that clinical pharmacists are responsible for changing medication prescriptions that are prescribed in the injectable dosage to oral dosage form for patients that are able to take oral form. |
| Stop order | This alert is generated to notify the physician that to proceed with the discontinuation or modification of this medication order the correct encounter number must be selected.  This alert is specific for medications prescribed for patients in the emergency department, which have a start time of within 24 hours later (physician orders the medication to be started within the next 24 hours).  The alert prompts the physician to select the correct encounter number for the patient, and this number is related to eligibility of patient to receive treatment at King Saud University Medical City. Thus, this alert is especially relevant for patients arriving to the emergency department, and receive treatment with a temporary electronic health record but are otherwise not eligible to receive treatment at King Saud University Medical City. | Inpatient (Only Emergency Department) | It began to be generated from May 2016. |
| Vincristine alert | Prevent users from entering orders for vincristine greater than 2mg. | Inpatient |  |

Appendix B: Criteria and parameters for assessment of appropriateness of alert display and appropriateness of alert override.

General information from the electronic health record of the patient is needed to assess alert for appropriateness of alert display and appropriateness of alert override as well as specific information according to the alert type. For each randomised alert, chosen reason from the drop-down menu and the free text documented to justify the override were examined. The patient’s electronic health record was accessed by the researchers. In cases where the electronic health record could not be accessed, for example this could be because the patient was no longer eligible to receive treatment at King Saud University Medical City, or if the patient passed away, the alert could not be assessed for appropriateness of display or override. The general information looked up for each medication-related alert was the date the medication was prescribed, and past medication history. The laboratory tests’ results were also accessed, dating back as was necessary to have baseline values in addition to past medical tests and vaccination history. Documentation of allergies (allergies to medications, or to food, or to elements from the environment such as dust or pollen) or of glucose 6-dehydrogenase deficiency syndrome on the electronic health record were also checked. The LexiComp ® (2018, 2019) software database was accessed for information regarding drug-drug interactions, therapeutic equivalents, and pharmacologic class member information and to cross analyse the documented allergies to medication, food, or environment with the medication ordered that generated the alert.

Specific for the alerts generated from the outpatient, the amount of medication dispensed was checked in addition to other medications ordered in the same clinic visit, as well as previous medication refill date. Specific information sought for the alerts generated from the inpatient department was the date of patient admission, the hospital ward admitted in, and the time the medication was ordered. The physician notes documented in the electronic health record were also read for any instructions to discontinue a medication or increase or decrease a dose or other adjustments in the patient’s medication regimen. Medication orders for patients in the inpatient wards are valid for one to two days, have a start date as well as an end date, and the start time will be set by the system. The physician can manipulate the start time, (the administration time) and this is especially relevant for antibiotics or medications needed immediately. The electronic health record and physicians’ notes were compared with the active medication list to determine which medications the patient had refilled/dispensed, and which were discontinued. The table below contains the criteria parameters assessed for each alert type to determine the appropriateness of alert display and the appropriateness of overriding the alert.

| Alert type | Criteria and parameters assessed | |
| --- | --- | --- |
| Drug  duplicate  Outpatient department | **Additional information needed** | The patient’s current medication list was examined for the medication that generated the alert, the previous prescription date was documented, and the quantity dispensed calculated. The medication list was also searched for medications from the same pharmacologic class, or another pharmacologic class but the same therapeutic class as the medication that generated the alert. The drug duplicate alert would be generated even if a medication had been discontinued. For example, the order of a topical dosage form of one of the non-steroidal anti-inflammatory medications would generate an alert if the patient’s medication list contained an oral or injectable form of non-steroidal anti-inflammatory. Therefore, the electronic health record was analysed thoroughly for the indication as well as the duration of the topical dosage form. An indication as osteoarthritis, or rheumatoid arthritis was noted as appropriate to have two dosage forms of the non-steroidal anti-inflammatory medication. |
|  | **Alert display** | **The alert display was assessed as appropriate if:**   - the patient still had enough quantity of medication based on the date of the previous refill, - the prescribed medication was in the same pharmacological class as another medication on the patient’s medication list - there was no documented change in electronic health record regarding any adjustment in medication strength, frequency, or dosage adjustment   **The alert display was assessed inappropriate if**:   - the patient did not have enough medication based on calculation of available medication expected since last documented refill date - the medication list did not contain a medication from the same pharmacologic class or a therapeutic equivalent - there was a documented change in electronic health record regarding any adjustment in medication strength, frequency, or dosage adjustment - the medication generating the alert is in the same pharmacological class as a medication on the patient’s mediation list; that is no longer available in the hospital or out of stock - the medication generating the alert is in the same pharmacological class as a medication on the patient’s mediation list that has however been discontinued and the patient not receiving it. |
|  | **Alert override** | **The alert override was assessed appropriate if:**   - the patient did not have enough medication based on calculation of available medication expected since last documented refill date - the prescribed medication was not in the same pharmacological class as another medication on the patient’s medication list - there was a documented change in electronic health record regarding any adjustment in medication dosage, strength, frequency - The medication generating the alert is in the same pharmacological class as a medication on the patient’s mediation list; that is no longer available in the hospital or out of stock - medication generating the alert is in the same pharmacological class as a medication on the patient’s mediation list that has however been discontinued and the patient not receiving it - The patient’s medication list did contain the same medication, which is causing alert generation, but in a different dosage form (for example oral and topical dosage forms of the same medication). - The prescribed medication was identical to another medication prescribed but in a different strength to complete the prescription for a patient. For example, prescription of 5mg warfarin tablet to be taken orally once a day and a prescription of 10mg warfarin tablet to be taken orally once a day because the patient needs to take a dose of 15mg warfarin once a day.   **The alert override was assessed inappropriate if:**   - the patient had enough medication based on calculation of available medication expected since last refill date - the current active medication list did contain a medication from the same pharmacologic class or a therapeutic equivalent and the patient would be administered the two medications simultaneously. - There was no documented change in electronic health record regarding any adjustment in medication dosage form, strength, or frequency - The prescribed medication was an identical to another medication prescribed but in a different strength to complete the prescription for a patient. For example, prescription of 5mg warfarin tablet to be taken orally once a day and a prescription of 10mg warfarin tablet to be taken orally once a day because the patient needs to take a dose of 15mg warfarin once a day. |
| Drug  Duplicate  Inpatient department | **Additional information needed** | The patient’s current medication list was searched for the medication that generated the alert, the previous prescription’s time and date was documented. The medication list was also searched for medications from the same pharmacologic class, or another pharmacologic class but the same therapeutic class as the medication that generated the alert. The physician notes documented in the electronic health record were also read for any instructions to discontinue a medication or increase or decrease the dose of medication. Prescribing a topical dosage form of one of the non-steroidal anti-inflammatory medications would generate a drug duplicate alert if the patient’s medication list contained an oral or injectable form of non-steroidal anti-inflammatory medication. Therefore, the electronic health record was analysed thoroughly for the indication as well as the duration of the topical dosage form. An indication as osteoarthritis, or rheumatoid arthritis was noted as appropriate to have two dosage forms of the non-steroidal anti-inflammatory medication. |
|  | **Alert display** | **The alert display was assessed as appropriate if:**   - The same medication order was placed twice in the same day - The prescribed medication was in the same pharmacological class as another medication on the patient’s medication list - The prescribed medication was an identical to another medication prescribed but in a different strength to complete the prescription for a patient. For example, prescription of 5mg warfarin tablet to be taken orally once a day and a prescription of 10mg warfarin tablet to be taken orally once a day because the patient needs to take a dose of 15mg warfarin once a day.   **The alert display was assessed inappropriate if**:   - the order was placed within the 24-hour interval of the previous medication order but ordered for the new day. - the medication list did not contain a medication from the same pharmacologic or a therapeutic equivalent |
|  | **Alert override** | **The alert override was assessed appropriate if:**   - the order was placed as a future order (within the 24-hour interval of the current order) to start after the current one is finished - the prescribed medication was not in the same pharmacological class as another medication on the patient’s medication list.   **The alert override was assessed inappropriate if:**   - the order was placed less than the 24-hour interval of the previous medication, and was not a future order - the medication list contained a medication from the same pharmacologic class or a therapeutic equivalent that was in necessary and exposed the patient to risk of side effects. |
| Drug  Interaction  Outpatient department and Inpatient department | **Additional information needed** | The medications on the current medication list were analysed for interactions with the medication that caused the alert using the Cerner Multum® and the LexiComp® (2018 and 2019) database software. |
|  | **Alert display** | **The alert display was assessed as appropriate if the medication causing the drug interaction alert:**   - interacted with one of the medications on the current medication list.   **The alert was assessed as not appropriately displayed if:**   - the medication did not interact with a medication on the current medication list - the medication interacted with a medication in the electronic health record but this medication was not on the patient’s active medication list (a discontinued medication) |
|  | **Alert override** | **The alert override was assessed appropriate if the medication causing the drug interaction alert:**   - did not interact with a medication on the current medication list - the medication interacted with a medication in the electronic health record but not on active medication list (a discontinued medication) - was cross checked with LexiComp® and determined to be an interaction of rating A, B, C or X with a medication in the electronic health record but not on active medication list (a discontinued medication) - was cross checked with LexiComp® and determined to be an interaction of rating A or rating B (the meaning of the ratings is in Appendix N). - was cross-checked using LexiComp® and determined to be rating C; and there was enough evidence in the electronic health record indicating necessary monitoring measures had been undertaken - was cross-checked using LexiComp® and determined to be rating C; and there was enough evidence in the electronic health record indicating this combination of medications was necessary and there were no suitable alternatives and the patient had tolerated it in the past - was cross checked using LexiComp® and determined to be rating D and there was enough information in the electronic health record demonstrating there was no other suitable alternative (either due to allergies, or more severe drug interactions) - was topically applied otic or optic preparation - was topically applied as ointment or cream for pain for short duration and no safer alternative was available   **The alert override was assessed not appropriate if the medication causing the drug interaction alert:**   - Was cross-checked using LexiComp® and determined to be rating C; and there was enough evidence in the electroni health record indicating necessary monitoring measures had been not been undertaken - Was cross-checked using LexiComp® and determined to be rating C; and there was enough evidence in the electronic health record indicating this combination of medications was not necessary and there were suitable alternatives and the patient had not tolerated it in the past - cross-checked using LexiComp® and determined to be rating D and there was enough information in the electronic health record demonstrating there were other safer alternatives - cross-checked using LexiComp® and determined to be interaction of rating X. |
| Dose-range  Outpatient and inpatient department | **Additional information needed** | The dose-range alert would be generated to alert the prescriber to check the dose of the medication being ordered. Thus, if a medication was prescribed outside these thresholds an alert would be generated. The recommended dose range of the prescribed medication that caused the alert generation was cross-checked using LexiComp® (2018 and 2019) database software. For medications used for indications that were off-label and not in the monograph of LexiComp® (2018 and 2019), UpToDate® (2018 and 2019) was searched for the recommended dose range. |
|  | **Alert display** | **The alert display was assessed as appropriate if the medication causing the dose-range alert was:**   - prescribed above or below the recommended reviewed dose range modified in Cerner Multum® and in the drug monograph in LexiComp® (2018 and 2019) database software. - a medication to be prescribed according to body weight in kilograms as per Cerner Multum ® but the patient’s weight was not documented in the electronic health record as part of vital signs, physician progress notes, or patient’s growth chart - prescribed above or below the recommended dose range in the drug and disease monograph from UpToDate® for an off-label indication - dependent on creatinine clearance values, however up to date serum creatinine laboratory results were not available   **The alert display was assessed as inappropriate if the medication causing the dose-range alert was:**   - prescribed within the recommended reviewed dose range modified in dose range in the recommended dose range of Cerner Multum® drug monograph |
|  | Alert override | **The alert override was assessed to be appropriate if the medication causing the dose-range alert was:**   - prescribed within the recommended reviewed dose range modified in Cerner Multum® drug monograph and dose range in the drug monograph in LexiComp® (2018 and 2019) or within the range confirmed - prescribed within the recommended reviewed dose range modified in dose-range according to body weight in kilograms as per Cerner Multum ®. The patient’s weight would sometimes be missing from the vital sign documented but be documented in the physician progress notes. - prescribed within the recommended reviewed dose range in the drug monograph in range of Cerner Multum® according to calculated creatinine clearance. The creatinine clearance was also calculated using Cockgroft and Gault equation for adults with renal impairment, and LexiComp® (2018, 2019) for creatinine clearance in paediatrics. - prescribed within the recommended dose range in the drug and disease monograph from UpToDate (2018 and 2019) for an off-label indication and there was enough information in the electronic health record to determine the justification for off-label use.   **The alert override was assessed to be inappropriate if the medication causing the alert was:**   - prescribed out of (higher or lower than) the recommended reviewed dose range in the drug monograph of Cerner Multum® and in the drug monograph in LexiComp® (2018 and 2019) - prescribed outside (higher or lower than) the dose-range according to body weight in kilograms in the drug monograph in LexiComp® - prescribed outside (higher or lower) the recommended dose range according to calculated creatinine clearance using Cockgroft and Gault equation for adults with renal impairment, and LexiComp® calculator (2018, 2019) for creatinine clearance in adults and paediatrics. |
| Drug allergy  Outpatient and inpatient department | **Additional information needed** | This drug allergy alert appears if an allergy is documented in the patient’s electronic health record. The electronic health record was reviewed for documented allergies to medication, food, or environment or insect as well as the severity of the allergy and reaction type. The date of documentation of this allergy in the electronic health record was also reviewed. The electronic health record was reviewed for evidence if anti-allergenics were administered/dispensed. For example, the epinephrine autoinjector or antihistamine (to be administered orally, topically or injectable) as anti-allergenics.  The medication causing drug-allergy alert generation was cross-checked using LexiComp® (2018 and 2019) database software. The King Saud University Medical City formulary was also reviewed in the instance the of vaccines, to determine if the preparation available and examine list of constituents. |
|  | **Alert display** | **The drug-allergy alert was assessed as appropriately displayed if:**   - The patient had a documented allergy in the electronic health record to the medication for which the alert was generated; or to a medication that was in the same pharmacological class; or to a constituent of the medication causing the generation of the alert.   **The drug-allergy alert was assessed as inappropriately displayed if:**   - the patient did not have a documented allergy to the medication causing the alert, nor to a medication in the same pharmacological class nor to a constituent of the medication causing the alert to be generated |
|  | **Alert override** | **The drug-allergy alert was assessed as appropriately overridden if:**   - the patient did not have a documented allergy to the medication causing the alert generation, or any its constituents, nor to a medication in the same pharmacological class as the medication for which the alert was generated - the patient had a documented mild allergy to the medication causing the alert; or to a medication in the same pharmacological class; or to a constituent of the medication causing the alert to be generated. The electronic health record was reviewed for evidence of the severity of the allergy and presence of symptoms. - The patient had a documented allergy in the electronic health record to the medication causing the alert; or to a medication in the same pharmacological class; or to a constituent of the medication causing the alert to be generated. However, the allergic reaction and severity were not documented. When cross checked with LexiComp® the identified risk level was A or B or C. The meaning of the risk levels for allergies to medication, food, and constituents of medication are in **Appendix N**. There was enough information to determine the patient had received the medication on an earlier occasion with no sequala, and that no feasible alternatives were available. - The mediation causing the alert was a topical medication. The patient had a documented allergy in the EHR to the oral or injectable form of the medication causing the alert, OR to a medication in the same pharmacological class OR to a constituent of the medication causing the alert to be generated. However, the allergic reaction and severity were not documented. When cross checked with LexiComp® the identified risk level was D or X. There was enough information in the electronic health record to determine the patient had received the topical medication before with no sequala. - The medication mediation causing the alert was an oral or injectable medication. The patient had a documented allergy in the electronic health record to the oral or injectable form of the medication causing the alert; or to a medication in the same pharmacological class; or to a constituent of the medication causing the alert to be generated. However, the allergic reaction and severity were not documented. When cross checked with LexiComp® the identified risk level was D or X. There was enough information in the electronic health record to determine the patient had received the oral or injectable medication before with no sequala, and that no feasible alternatives were available.   **The drug-allergy alert was assessed as overridden inappropriately if:**   - The patient had a documented severe allergy to the medication causing the alert; or to a constituent of the medication causing the alert to be generated; or to a medication in the same pharmacological class. The electronic health record was reviewed for evidence of the severity of the allergy and presence of symptoms. - The mediation causing the alert was an oral or injectable medication. The patient had a documented allergy in the electronic health record to the oral or injectable form of the medication causing the alert; or to a medication in the same pharmacological class; or to a constituent of the medication causing the alert to be generated. However, the allergic reaction and severity were documented as severe. When cross checked with LexiComp® the identified risk level was A, B, or C. There was enough information in the electronic health record to determine the patient had received the medication before with sequala. - The mediation causing the alert was an oral or injectable medication. The patient had a documented allergy in the electronic health record to the oral or injectable form of the medication causing the alert; or to a medication in the same pharmacological class; or to a constituent of the medication causing the alert to be generated. However, the allergic reaction and severity were documented as severe. When cross checked with LexiComp® the identified risk level was D or X. There was enough information in the electronic health record to determine other alternatives could have been chosen, even if the patient had received the medication before with sequala. |
| New drug allergy  Outpatient and inpatient department | **Additional information needed** | This is a reverse chronological alert referred to as ‘new drug allergy’ alert. It appears when the drug is prescribed, and then an allergy is documented on the electronic health record afterwards. The electronic health record was reviewed for the date of documented allergies to medication, food, or environment, or insect as well as the severity of the allergy and reaction type. The electronic health record was reviewed for evidence if anti-allergenics were administered/dispensed. For example, the epinephrine autoinjector or an antihistamine to be administered orally, topically or injectable.  The medication causing new drug allergy alert generation was cross-checked using LexiComp® (2018 and 2019) database software. The King Saud University Medical City formulary was also reviewed in the instance of vaccines, to determine the preparation available and examine list of constituents. |
|  | **Alert display** | **The new drug allergy alert was assessed as appropriately displayed if:**   - The patient had a documented allergy in the electronic health record to the medication causing alert generation; or to a medication that was in the same pharmacological class; or to a constituent of the medication causing the generation of the alert.   **The new drug allergy alert was assessed as inappropriately displayed if:**   - the patient did not have a documented allergy to the medication causing the alert, nor to a medication in the same pharmacological class nor to a constituent of the medication causing the alert to be generated. |
|  | **Alert override** | **The new drug allergy alert was assessed as appropriately overridden if:**   - the patient did not have a documented allergy to the medication causing the alert, nor to a medication in the same pharmacological class nor to a constituent of the medication causing the alert to be generated - The patient had a documented mild allergy to the medication causing the alert; or to a medication in the same pharmacological class; or to a constituent of the medication causing the alert to be generated. The electronic health record was reviewed for evidence of the severity of the allergy and presence of symptoms and were confirmed to be mild symptoms or without symptoms. - The patient had a documented allergy in the electronic health record to the medication causing the alert; or to a medication in the same pharmacological class; or to a constituent of the medication causing the alert to be generated. However, the allergic reaction and severity were not documented. When cross checked with LexiComp® the identified risk level was A or B or C. The meaning of the risk levels for allergies to medication, food, and constituents of medication are in **Appendix** N. There was enough information in the electronic health record to determine the patient had received the topical medication before with no sequala. - The mediation causing the alert was a topical medication. The patient had a documented allergy in the electronic health record to the oral or injectable form of the medication causing the alert; or to a medication in the same pharmacological class; or to a constituent of the medication causing the alert to be generated. However, the allergic reaction and severity were not documented. When cross checked with LexiComp® the identified risk level was D or X. There was enough information in the electronic health record to determine the patient had received the topical medication before with no sequala.   **The new allergy-drug alert override was assessed as inappropriate if:**   - The patient had a documented severe allergy to the medication causing the alert; or to a medication in the same pharmacological class; or to a constituent of the medication causing the alert to be generated. The electronic health record was reviewed for evidence of the severity of the allergy and presence of symptoms. - The mediation causing the alert was an oral or injectable medication. The patient had a documented allergy in the electronic health record to the oral or injectable form of the medication causing the alert; or to a medication in the same pharmacological class; or to a constituent of the medication causing the alert to be generated. However, the allergic reaction and severity were documented as severe. When cross checked with LexiComp® the identified risk level was A, B, or C. There was enough information in the electronic health record to determine the patient had received the medication before with sequala. - The mediation causing the alert was an oral or injectable medication. The patient had a documented allergy in the electronic health record to the oral or injectable form of the medication causing the alert; or to a medication in the same pharmacological class; or to a constituent of the medication causing the alert to be generated. However, the allergic reaction and severity were documented as severe. When cross checked with LexiComp® the identified risk level was D or X. There was enough information in the electronic health record to determine other alternatives could have been chosen, even if the patient had received the medication before with sequala. |
| Egg-allergy alert  Outpatient and inpatient department | **Additional information needed** | This egg allergy alert appears if an egg allergy or egg-derived product allergy is documented in the patient’s electronic health record. The electronic health record was reviewed for documented allergies to egg-allergy, medication or food, as well as the severity of the allergy and reaction type. The date of the documentation of the allergy was also reviewed. The electronic health record was reviewed for evidence if anti-allergenics were administered/dispensed. For example, the epinephrine autoinjector or an antihistamine to be administered orally, topically or injectable.  The medication causing egg-allergy alert generation was cross-checked using LexiComp® (2018 and 2019) database software. The King Saud University Medical City formulary was also reviewed in the instance of vaccines, to determine the preparation available and examine list of active constituents. |
|  | **Alert display** | **The egg-allergy alert was assessed as appropriately displayed if:**   - The patient had a documented egg-allergy in the electronic health record and the medication for which the alert was generated had a constituent of egg protein or derivative.   **The egg-allergy alert was assessed as inappropriately displayed if:**   - The patient did not have a documented egg allergy in the electronic health record (from the sample of alerts analysed this did not occur) - The medication causing the alert to be generated did not have egg or egg-derived product in its constituent list   (In the sample of alerts analysed and assessed there was no instance of this alert being displayed inappropriately). |
|  | **Alert override** | **The egg-allergy alert was assessed as appropriately overridden if:**   - The patient did not have a documented egg-allergy - The medication causing the alert to be generated did not have egg derived product in its constituent list - The patient had a documented egg-allergy, but the medication causing the alert to be generated did not have egg or egg-derived product listed as part of its constituents - The patient had a documented egg-allergy in the electronic health record however the allergic reaction and severity were not documented. The medication for which the alert was generated did have egg or egg derived product listed as part of its constituents, and when cross checked with LexiComp® the identified risk level was A or B or C or X. The meaning of the risk levels for egg allergies with medication are in **Appendix N**. There was enough information to determine that the patient had received this medication before with no sequala. - The patient had a documented egg-allergy in the electronic health record; however, the allergic reaction and severity were not documented. The vaccine causing the alert to be generated did have egg or egg derived product as part of its constituents, and when cross checked with LexiComp® the identified risk level was A, B, or C. The meaning of the risk levels for egg allergies with medication are in **Appendix N**. There was enough information to determine that the patient had received this medication with no sequala. - The patient had a documented egg-allergy in the electronic health record; however, the allergic reaction and severity were not documented. The vaccine causing the alert to be generated did have egg or egg-derived product in its constituents, and when cross checked with LexiComp® the identified interaction risk level was D or X. The meaning of the risk levels for egg allergies with medication are in **Appendix N**. There was enough information to determine that the patient had received this medication with no sequala, and there were no other suitable alternatives to be used.   **The egg-allergy alert was assessed as inappropriately overridden if:**   - The patient had a documented egg-allergy in the electronic health record; however, the allergic reaction and severity were not documented. The vaccine causing the alert to be generated did have egg or egg derived protein as part of its constituents, and when cross checked with LexiComp® the identified risk level was A. The meaning of the risk levels is in **Appendix N**. There was enough information to determine that the patient had received this before medication with sequala and needed anti-allergic medication (there were none in our sample found to be assessed like this). - The patient had a documented egg-allergy in the electronic health record; however, the allergic reaction and severity were not documented. The vaccine causing the alert to be generated did have egg or egg-derived product in its constituents, and when cross checked with LexiComp® the identified risk level was D or X. The meaning of the risk levels for egg allergies with medication are in **Appendix N**. There was enough information to determine that the patient had previously received this medication with sequala and needed anti-allergic medication and there were other suitable safer alternatives to be used (In the sample of alerts analysed and assessed there was no instance of this alert being overridden inappropriately). |
| Glucose-6 phosphate dehydrogenase deficiency syndrome  Outpatient and inpatient department | **Additional information needed** | This alert is generated for medication that will not be metabolised effectively if taken by a patient suffering from glucose-6 phosphate dehydrogenase deficiency syndrome.  The electronic health record was reviewed for confirmation of glucose-6 phosphate dehydrogenase deficiency syndrome  syndrome. The medication was cross-checked in LexiComp® and the monograph reviewed regarding administration for patients with glucose-6 phosphate dehydrogenase deficiency syndrome. |
|  | **Alert display** | **The glucose-6 phosphate dehydrogenase** **deficiency syndrome**  **syndrome alert was assessed as appropriately displayed if:**  The patient had confirmed glucose-6 phosphate dehydrogenase deficiency syndrome.   - The medication causing the alert was to be used with caution or contraindicated to be administered to patients with glucose-6 phosphate dehydrogenase deficiency syndrome   **The glucose-6 phosphate dehydrogenase syndrome alert was to be assessed as inappropriately displayed if:**   - The patient did not have glucose-6 phosphate dehydrogenase deficiency syndrome. (In the sample of alerts analysed and assessed there was no instance of this alert being generated inappropriately.) |
|  | **Alert override** | **The alert was assessed as appropriately overridden if:**   - There was enough evidence in the electronic health record to confirm the patient tolerated this medication before, and there was no other suitable alternative.   **The alert was assessed as inappropriately overridden if**:   - the medication was to be used with caution or contraindicated to be administered to patients with glucose-6 phosphate dehydrogenase deficiency syndrome but there was no evidence that the medication had been tolerated with no sequala. (In the sample of alerts analysed and assessed there was no instance of this alert being overridden inappropriately.) |
| Switch medication from injectable dosage form to oral dosage form.  Inpatient and outpatient | **Additional information needed** | This alert is generated to alert the physician to switch the dosage form ordered from injectable to oral. If one of the medications on the patients list is orally administered the one prescribed in injectable form will trigger the alert generation. However, some medications are tolerated to be crushed and inserted via enteral nutrition or via nasogastric tube. The electronic health record was reviewed to determine if the patient could or could not tolerate administered oral medications (for example was fasting before surgery or recovering post-surgery) or had issues with swallowing. The medication (causing the alert generation) was checked using LexiComp® 2018 and 2019. The King Saud University Medical City medication formulary was also checked, to determine availability of oral dosage form of the prescribed medication. |
|  | **Alert display** | **The alert was assessed as appropriately displayed if:**   - The patient could tolerate administration of oral dosage form and the medication was available in oral dosage forms   **The alert was assessed as inappropriately displayed if:**   - The patient was currently on physicians’ instructions to not take food, drink or medications orally. - The medication was not available in King Saud University Medical City as oral dosage form - Due to the indication (reason the medication was prescribed), the medication was to be administered injectable route rather than oral route. This was determined from the electronic health record and physician’s notes, and checked with the medication monograph from LexiComp ® (2018 and 2019) |
|  | **Alert override** | **The alert was assessed as appropriately overridden if:**   - The patient was currently on physicians’ instructions to not take food, drink or medications by mouth (nothing by the oral route) - The medication was not available in in King Saud University Medical City as oral dosage form, available only injectable dosage form - Due to the indication (reason the medication was prescribed), the medication was to be administered by injectable route rather than oral route. This was determined from the electronic health record and physician’s notes and checked with the medication monograph from LexiComp ® (2018 and 2019). - An oral alternative is not available in King Saud University Medical City formulary.   **The alert override was assessed to be inappropriate if:**   - The patient could tolerate oral medications and the medication’s oral dosage form was available in King Saud University Medical City formulary. - The patient could tolerate intake of oral medications and an oral alternative was available in King Saud University Medical   formulary. |
| Documentation of height and weight  Inpatient and outpatient | **Additional information needed** | If patient’s height and weight information are missing from the electronic health record, or patient’s growth chart, for a patient aged 12 years or less, this alert will be generated.to notify the physician to check the dose of the medication prescribed. The patient’s EHR was reviewed for the documentation of the patient’s weight and height. LexiComp® (2018 and 2019), UpToDate® (2018 and 2019) were searched for the recommended dose range of the medication that caused the alert to be generated. To assess the appropriateness of the alert override, the electronic health record was reviewed, for evidence of documentation of patient’s weight or height on the date the medication was prescribed, including the during documentation of vital signs, patient’s growth chart, and physician’s notes. |
|  | **Alert display** | **The alert was assessed as appropriately displayed if:**   - There was no documentation of patient’s weight and height in the electronic health record. - The dosing of medication for which the alert was generated depends on weight or body surface area calculation.   **The alert was assessed as inappropriately displayed if:**   - there was documentation of patient’s weight and height in the electronic health record as part of patient demographic information - The dosing of the medication for which the alert was generated does not depend on patient weight or calculation of body surface area. |
|  | **Alert override** | **The alert was assessed as appropriately overridden if:**   - The dosing of the medication that generated the alert does not depend on patient weight or calculation of body surface area. - The dosing of the medication that generated the alert does depend on patient’s weight, and upon review of the patient’s electronic health record, the updated weight was documented as part of vital signs or in the physician’s notes. The recommended dose of medication for which the alert was generated was checked through Lexicomp® (2018 and 2019). The dose of medication ordered by the physician was found to be within the recommended dose range.   **The alert override was assessed to be inappropriate if:**   - The dosing of the medication that generated the alert does depend on patient’s weight, and upon review of the patient’s electronic health record, the updated weight was documented as part of vital signs or in the physician’s notes. The recommended dose of medication for which the alert was generated was checked through the drug monograph of Lexicomp® (2018 and 2019). The dose of medication prescribed by the physician was found to be out of (higher or lower) than the recommended dose range. - The dosing of the medication that generated the alert does depend on patient’s weight, and upon review of the patient’s electronic health record, the updated weight was not documented as part of vital signs or in the physician’s notes. The recommended dose of medication for which the alert was generated was checked through the drug monograph Lexicomp® (2018 and 2019). The dose of medication prescribed by the physician was found to be prescribed out of (higher or lower) than the recommended dose range. |
| Vincristine dosage alert  Inpatient department | **Additional information needed** | The vincristine dose alert would be generated if the prescriber ordered more than 2mg, the reference for this dose-threshold was (Cerner Millenium, Multum®). The patient’s body surface area was calculated with the LexiComp calculator. From the patient’s electronic health record, the patient’s weight in kilograms was noted, and in case not documented in the electronic health record, the physician notes, laboratory results, and imaging results was reviewed to determine the indication the for which patient was receiving the vincristine (which type of malignancy). The recommended dose according to the indication was cross-checked using LexiComp® (2018 and 2019) database software and UpToDate® (2018 and 2019) database software. |
|  | **Alert display** | **The vincristine-dosage alert was assessed as appropriately displayed if:**   - Vincristine had been prescribed in a dose more than 2mg   **The vincristine-dosage alert was assessed as inappropriately displayed if:**   - Vincristine had been prescribed in a dose of 2mg or less |
|  | **Alert override** | **The vincristine-dosage alert was assessed as appropriately overridden if:**   - Vincristine had been prescribed in a dose of 2mg or less - Vincristine had been prescribed in a dose of greater than 2mg; however, upon review of the electronic health record there was enough information documented and the dosage was justified. The dose was within recommended range suitable for the patient and for the indication as per LexiComp® (2018 and 2019) and UptoDate® 2018 and 2019) - Vincristine was ordered but with-held from administration and the patient did not receive it   **The vincristine-dosage alert was assessed as inappropriately overridden if:**   - The targeted vincristine dose within the chemotherapeutic regimen was not documented - There was not enough information in the electronic health record to justify the targeted vincristine dose within the chemotherapeutic regimen - There was enough information in the electronic health record to determine the dose above 2mg was not justifiable based on patient’s indication, or BSA, as in the Lexi-Comp® (2018 and 2019) and UptoDate® monographs. |
| Stop order  Inpatient | **Additional information needed** | This alert is generated to indicate that this order will be ordered once and not repeated. The alert type was activated in May 2016.  The purpose the alert serves is to prevent repetition of prescription of medication. It is generated when patients come to the emergency department and are non-eligible to open a patient file and record. They are given temporary medical record numbers. For this reason, there is no electronic health record to access and assessing appropriateness of alert display and alert override during the pilot arm prove to not be possible, therefore this alert was not included for assessment in the study arm. |
|  | **Alert display** | Assessment not carried out |
|  | **Alert override** | Assessment not carried out |

Appendix C: Steps of assessment of appropriateness of alert display and alert override for each alert type

General process of seeking patient related information from the electronic health record and pharmacy records during assessment of appropriateness of alert display and alert override.

The overridden medication related alert (randomised for assessment) and the available alert information as in appendix A

Access the patient’s electronic health record and read the progress notes, as well as documentation of any allergies.

Search for the medication prescription that caused the alert to be generated and the progress notes according to date of alert.

Determine the active medication list from pharmacy records that the patient was taking for that specified time

Information sought from electronic health record specific for alert from outpatient department

Information sought from the electronic health record specific for alert from inpatient department

The date the patient was admitted, which hospital ward, the time the medication was ordered, and the duration.

The amount of medication dispensed and the previous refill date (if applicable).

Assessment of drug duplicate alert display:

Box One

Outpatient: Does the patient have enough of this medication at home?

Inpatient: Was this same order placed twice within the same 24 -hour period?

Yes or No Proceed to next question

Box Two

Outpatient and inpatient: Is the prescribed medication in the same pharmacological class as another medication on patient’s medication list?

Yes or No Proceed to next question

Box Three

Outpatient and inpatient: Was there a documented prescribed change in the electronic health record regarding any adjustment in medication strength, frequency, dosage or dosage form?

If the answer to all of the questions in box one, two, or three was no, the alert was assessed as displayed inappropriately.

If the answer to any of the questions in box one, two, or three was yes, the alert was assessed as displayed appropriately.

Assessment of drug-duplicate alert override:

Outpatient: Is a refill of the prescribed medication due?

Inpatient: Is this medication prescribed twice in the same 24-hour period?

Box One

Yes or no; proceed to next question

Outpatient and inpatient: Is the prescribed medication in the same pharmacological class as another medication on patient’s medication list?

Box Two

Yes or no; proceed to next question

Box Three

Outpatient and inpatient: Was this order to prescribe a change in the electronic health record regarding any adjustment in medication strength, frequency, dosage or dosage form?

Yes or No Proceed to next question

Outpatient and inpatient: Was this order to prescribe a change in the strength of the medication, necessitating two separate orders to complete the required strength?

Box Four

If the answer to any of the questions in box one, two, three, or four was ‘no’ the alert was assessed as overridden inappropriately.

If the answer to the questions in box one, two, three and four was ‘yes’, the alert was assessed as overridden appropriately.

Assessment of drug interaction alert display:

Outpatient and inpatient:

Does the medication causing the alert interact with any medication on the active medication list?

Box One

Yes

No

If the answer to the question in box one was ‘no’, the alert was assessed as being displayed inappropriately.

If the answer to the question in box one was ‘yes’, the alert was assessed as being displayed appropriately.

Assessment of drug interaction alert override

Outpatient and inpatient:

Does the medication causing the alert interact with any medication on the active medication list?

The alert was assessed as overridden appropriately.

No

Yes

Yes

Was this medication an otic or opthalmic preparation for short term use?

Was this medication a topical preparation for short term use?

The alert was assessed as overridden appropriately.

No

Upon cross checking with Lexi-Comp ® this interaction was determined to be an interaction of rating A, or B, or C, or D, or X. The next page details the assessment of drug interaction alert according to rating.

Assessment of drug interaction alert override according to rating:

Upon cross checking with the Lexi-Comp was this interaction determined to be an interaction of rating A or rating B?

Yes

Appropriate override

No

Inappropriate override

Upon cross checking with the Lexi Comp this interaction it was determined to be of rating X.

Yes

No

Was there enough evidence in the electronic health record to indicate that necessary monitoring measures had been undertaken?

Yes

Upon cross checking with the Lexi-Comp was this interaction determined to be of rating C?

No

Yes

No

Inappropriate override

Upon cross checking with the Lexi-Comp was this interaction determined to be of rating C or D?

Appropriate override

Yes

Yes

Appropriate override

Was there was enough evidence in the electronic health record to indicate that this combination of medications was necessary?

No

Inappropriate override

Yes

Was a suitable alternative available in King Saud University Medical City formulary and in stock?

(Suitable for the patient, according to age and allergy status and did not have other or more severe drug interactions with other concurrent medications).

Appropriate override

No

Assessment of dose range alert display:

Box one

Inpatient and outpatient:

Was the dosage medication causing generation of dose range alert prescribed above or below the recommended dose range as checked in Cerner Multum® database?

Appropriately alert display

Yes

No or not applicable

Yes

Inappropriate alert display

Inpatient and outpatient:

Was the dosage medication causing generation of dose range alert to be prescribed according to body weight?

Box two

Yes

Was the recent body weight documented in the electronic health record?

No

Appropriate alert display

No or not applicable

Box three

Inpatient and outpatient:

Was the dosage medication causing generation of dose range alert prescribed above or below the recommended dose range as checked in UpToDate® for an off-label indication?

Yes

Appropriate alert display

No or not applicable

Inpatient and outpatient:

Was the dosage medication causing generation of dose range alert to be prescribed according to serum creatinine level but there was no documentation of recent serum creatinine level?

Yes

Appropriate alert display

Box four

No or not applicable

If the answer to the questions in boxes one through four was ‘no’, the alert was assessed as displayed inappropriately.

Assessment of dose range alert override in inpatient and outpatient:

Yes

Box one

Was the dosage medication causing generation of dose range alert prescribed above or below the recommended dose range as checked in the revised Cerner Multum® database?

Inappropriate alert override

No or not applicable

Appropriate alert override

Yes

Was the recent body weight documented in the electronic health record?

Box two

Was the dosage medication causing generation of dose range alert to be prescribed according to body weight?

Inappropriate alert override

No

Yes,the supporting evidence was enough

No or not applicable

Was the dosage prescribed above or below the recommended dose range as checked in UpToDate® with enough information documented in the EHR supporting this use?

Box three

Was the dosage of the medication causing generation of dose range alert prescribed for an off-label indication?

Appropriate alert override

Inappropriate alert override

No, the supporting evidence was not enough

No or not applicable

Yes, the supporting evidence was enough

Box four

Was the dosage of the medication causing generation of dose range alert to be prescribed according to serum creatinine level?

Appropriate alert override

Was there documentation of recent serum creatinine level, and upon calculation of creatinine clearance the prescribed dose was suitable for the patient?

Inappropriate alert override

No, the supporting evidence was not enough

Assessment of drug allergy alert display in inpatient and outpatient:

Was there a documented allergy in the patient’s electronic health record to the medication causing alert generation?

Appropriate alert display

Yes

Box one

No

Was there a documented allergy in the patient’s electronic health record to a medication in the same class as the medication causing alert generation?

Yes

Appropriate alert display

Box two

No

Yes

Was there a documented allergy in the patient’s electronic health record to a constituent of the medication causing alert generation?

Appropriate alert display

Box three

If the answer to the questions in boxes one through three was ‘no’, the alert was assessed as displayed inappropriately.

Assessment of drug allergy alert override in inpatient and outpatient:

Was there a documented allergy in the patient’s electronic health record to a constituent of the medication causing alert generation?

Was there a documented allergy in the patient’s electronic health record to a medication in the same class as the medication causing alert generation?

Was there a documented allergy in the patient’s electronic health record to the medication causing alert generation?

Yes

Yes

No

No

Yes

No

Box one

If the answer to questions in boxes one, two and three was ‘no’ the alert was assessed as overridden appropriately

How was the type and severity of the allergic reaction documented in the patient’s electronic health record?

Severe

Not documented

Was there enough information documented in the electronic health recordindicating the patient had taken the medication before?

Was it topical, ophthalmic, or an otic dosage form for short term use?

Mild or moderate

Yes

When cross checked with LexiComp® was the identified risk level either level A, level B, or level C?

No

Yes

No

Appropriate alert override

Inappropriate alert override

Inappropriate alert override

Yes

Was there enough information documented in the electronic health record that the patient had taken the medication before without sequala?

If there were no available alternatives please go to next page to continue this figure:

Continuation of figure: Assessment of drug allergy alert override in inpatient and outpatient:

Was there enough information documented in the electronic health record to show the patient did not have sequala, and there were no suitable alternatives?

No

Yes

Appropriate alert override

Inappropriate alert override

Assessment of new drug allergy alert display

Was there a documented allergy in the patient’s electronic health record to the medication causing alert generation?

No

Yes

Yes

Appropriate alert display

Appropriate alert display

Appropriate alert display

No

Was there a documented allergy in the patient’s electronic health record to a medication in the same class as the medication causing alert generation?

Was there a documented allergy in the patient’s electronic health record to a constituent of the medication causing alert generation?

No

If the answer to questions in boxes one, two and three was ‘no’ the alert was assessed as displayed inappropriately.

Assessment of new drug allergy alert override

Was there a documented allergy in the patient’s electronic health record to a constituent of the medication causing alert generation?

Was there a documented allergy in the patient’s electronic health record to a medication in the same class as the medication causing alert generation?

Was there a documented allergy in the patient’s electronic health record to the medication causing alert generation?

Yes

Yes

No

No

Yes

Box one

No

If the answer to questions in boxes one, two and three was ‘no’ the alert was assessed as overridden appropriately

How was the type and severity of the allergic reaction documented in the patient’s electronic health record?

Severe

Not documented

Mild or moderate

Was there enough information documented in the electronic health record indicating the patient had taken the medication before?

When cross checked with LexiComp® was the identified risk level either level A, level B, or level C?

Was it topical, ophthalmic, or an otic dosage form for short term use?

No

No

Yes

Inappropriate alert override

Appropriate alert override

Yes

Yes

Was there enough information documented in the electronic health record that the patient had taken the medication before without sequala?

Inappropriate alert override

Yes

Assessment of egg-allergy alert display

Inappropriate alert display

No

No

Yes

Was there a documented egg allergy in the patient’s electronic health record?

Inappropriate alert display

Did the medication that caused alert generation contain egg or egg-derived product?

Yes

Appropriate alert display

Assessment of egg-allergy alert override

No

Did the medication that caused alert generation contain egg or egg-derived product?

Appropriate alert override

Yes

Was there a documented egg allergy in the patient’s electronic health record?

No

Appropriate alert override

Yes

How was the type and severity of the allergic reaction documented in the patient’s electronic health record?

Yes

Not documented

Severe

Was there enough information documented in the electronic health record that the patient had taken the medication before without sequala?

Mild or moderate

Was there enough information documented in the electronic health record that the patient had taken the medication before without sequala?

When cross checked with LexiComp® was the identified risk level either level A, B, C, D or X?

Yes

No

No

Yes

No

Appropriate alert override

Inappropriate alert override

Yes

Was there another suitable available?

No

Assessment of egg-allergy alert override for vaccines

Appropriate alert override

No

Did the medication that caused alert generation contain egg or egg-derived product?

Yes

Appropriate alert override

No

Was there a documented egg allergy in the patient’s electronic health record?

Yes

How was the type and severity of the allergic reaction documented in the patient’s electronic health record?

Yes

Not documented

Mild or moderate

Severe

Was there enough information documented in the electronic health record that the patient had taken the medication before without sequala?

Was there enough information documented in the electronic health record that the patient had taken the medication before without sequala?

When cross checked with LexiComp® was the identified risk level either level A, B, C, D or X?

Yes

No

Yes

No

No

Was there another suitable available?

Was there enough information documented in the electronic health record that the patient had taken the medication before without sequala?

Inappropriate alert override

Yes

No

Appropriate alert override

Inappropriate alert override

Assessment of glucose 6 phosphate dehydrogenase syndrome alert display

Did the patient have confirmed (documented in the electronic health record) diagnosed glucose 6 phosphate dehydrogenase syndrome?

Inappropriate alert display

No

Yes

No

When checked in LexiComp® was the medication causing the alert to be generated found to be contraindicated for use in patients with glucose 6 phosphate dehydrogenase (or recommended to be used with caution)?

Inappropriate alert display

Yes

Appropriate alert display

Assessment of glucose 6 phosphate dehydrogenase syndrome alert override

Appropriate alert override

No

Did the patient have confirmed (documented in the electronic health record) diagnosed glucose 6 phosphate dehydrogenase syndrome?

Yes

No

When checked in LexiComp® was the medication causing the alert to be generated found to be contraindicated for use in patients with glucose 6 phosphate dehydrogenase (or recommended to be used with caution)

Appropriate alert override

Yes

Yes

Was there enough information documented in the electronic health record that the patient had taken the medication before without sequala?

Yes

No

Inappropriate alert override

Was there another suitable alternative available?

No

Appropriate alert override

Assessment of alert display ‘switch medication from injectable dosage form to oral dosage form’.

Was the medication available in the oral dosage form in King Saud University Medical City formulary and available in stock?

Appropriate alert display

Yes

Yes

Was the patient able to tolerate medication in the oral dosage form?

No

No

Inappropriate alert display

Assessment of alert override of type ‘switch medication from injectable dosage form to oral dosage form’.

Yes

Was the medication available in the oral dosage form in King Saud University Medical City formulary and available in stock?

Yes

In the electronic health record of the patient was it documented that the medication was prescribed in the injectable route rather than oral because of the indication?

Was the patient able to tolerate medication in the oral dosage form?

No

No

No

Inappropriate alert override

Yes

Appropriate alert override

Assessment of alert display of type ‘documentation of height and weight’ from inpatient and outpatient setting.

No

Was the patient’s age less than 12 years at the time of prescribing the medication? (After confirmation of the documented date of birth in the electronic health record).

Inappropriate alert display

Yes

Yes

Was the patient’s height and weight documented in the electronic health record (on the date of the prescription of the medication)?

Inappropriate alert display

No

Was the dose of the medication causing alert to be generated dependent on height, weight, or body surface area?

Inappropriate alert display

Yes

Assessment of alert override of type ‘documentation of height and weight’ from inpatient and outpatient setting.

Was the patient’s age less than 12 years at the time of prescribing the medication? (After confirmation of the documented date of birth in the electronic health record).

Appropriate alert override

No

Yes

No

Was the dosing of the medication causing the alert to be generated dependent on height or on weight, or on body surface area?

Appropriate alert override

Yes

Was the patient’s height and weight documented in the electronic health record (on the date of the prescription of the medication)?

No

Inappropriate alert override

Yes

Was the dose of the medication of the medication prescribed found to be within the recommended dose range (as checked in Cerner Multum® and LexiComp® 2018, 2019?

Yes

No

Inappropriate alert override

Appropriate alert override

Assessment of vincristine alert display in inpatient setting

No

Appropriate alert display

Was the dose of vincristine prescribed two milligrams or more?

Inappropriate alert display

Yes

Assessment of vincristine alert override in inpatient setting

Was the dose of vincristine prescribed two milligrams or more?

No

Appropriate alert override

Yes

No

Was there enough information documented in the electronic health record to justify the prescription of this dose?

Inappropriate alert override

Yes

No

Was the dose within the recommended range suitable for the patient and for the indication? (As per Cerner Multum®, and UptoDate® 2018 and 2019).

Inappropriate alert override

Yes

Appropriate alert override

Appendix D: Example of assessed alerts for appropriateness (from inpatient setting).

| Alert type | Alert date | Patient gender and year of birth | Alerted order | Reason  chosen for override from pull down menu | Free text entered for override  Reason | Consensus on alert display appropriate or not | Consensus on alert override appropriate or not |
| --- | --- | --- | --- | --- | --- | --- | --- |
| Drug  Duplicate alert | 18/09/2016 | M  1979 | Erythropoetin Alpha 4000units/0.4mL Preloaded syringe 4,000 unit(s), intravenous injection, once. | Physician Approved Override | No Overridden Reason Entered | Appropriate | Appropriate |
| Dose range alert | 15/12/2015 | M  2012 | Paracetamol 120mg/5mL syrup. 200 mg, orally every 6 hours, as needed for fever.  First Dose: 15/12/2015 | Physician Reviewed Drug Interaction | No Overridden Reason Entered | Appropriate | Appropriate |
| Drug allergy alert | 20/07/2016 | F  1942 | Amoxicillin-clavulanate 1gm, Oral tablet, orally every 12 hours for 14 days. (28 tablets).  No Refills. | No Overridden Reason Selected | No Overridden Reason Entered | Appropriate | Not appropriate |
| Glucose 6 phosphate dehydrogenase deficiency syndrome alert | 25/05/2016 | M  2014 | Dapsone 50 mg, oral.  Once a week, for 30 days.  No refills | Physician Approved Override | No Overridden Reason Entered | Appropriate | Appropriate |
| Vincristine alert | 04/09/2017 | F  1942 | Vincristine 2.3 mg = 2.3 vials, intravenous. One Injection.  For chemotherapy.  First Dose: 04/09/2017 | Physician Clinical Judgement | No Overridden Reason Entered | Appropriate | Not appropriate |

Appendix E: Example of assessed alerts for appropriateness (from outpatient setting).

| **Alert type** | **Alert date** | **Patient Gender and Year of birth** | **Alerted order** | **Reason**  **chosen for override from pull down menu** | **Free text entered for overridden Reason** | **Consensus on alert display appropriate or not** | **Consensus on alert override appropriate or not** |
| --- | --- | --- | --- | --- | --- | --- | --- |
| **Drug allergy alert** | 19/03/2017 | M  1951 | Ibuprofen 400 mg  Oral tablet, once daily.  As needed for pain.  For 10 days, no refills. | No Overridden Reason Selected | No Overridden Reason Entered | Appropriate | Not appropriate |
| **New drug allergy alert** | 26/4/2017 | F  1975 | Amoxicillin-clavulanate one gram oral tablets every 12 hour, for seven days. 14 tablets, no refills. | No Overridden Reason Selected | No Overridden Reason Entered | Appropriate | Not appropriate |
| **Documentation of height and weight alert** | 31/07/2017 | M  2006 | Acyclovir topical  1 application. Right eye, five times a day, X 14 days, no refills. | No Overridden Reason Selected | No Overridden Reason Entered | Appropriate | Appropriate |
| **Egg allergy alert** | 06/12/2015 | F  2014 | Influenza virus vaccine, inactivated. 0.25 mL intramuscular injection, once. First Dose: 06/12/2015 | Physician Reviewed drug interaction | No Overridden Reason Entered | Appropriate | Appropriate |
| **Drug interaction alert** | 14/12/2017 | F  1978 | Budesonide-formoterol Two puffs, inhalation two times a day for 180 days. No refills. | Physician approved override | No Overridden Reason Entered | Appropriate | Not Appropriate |

Appendix F: Alerts generated and overridden from inpatient setting.

| **Alert type** | **Alerts generated** | **Alerts overridden** | |
| --- | --- | --- | --- |
|  | Number of alerts | Number of alerts | (%) of this type |
| Dose range | 283,000 | 276,921 | 97.9 |
| Switch medication from injectable form to oral | 23,268 | 22,654 | 97.4 |
| Drug interaction | 258,813 | 251,722 | 97.3 |
| Vincristine | 17 | 16 | 94.1 |
| Duplicate drug | 2,170,376 | 2,011,958 | 92.7 |
| Drug allergy | 9,228 | 8,473 | 91.8 |
| Documentation of height and weight | 47,377 | 42,813 | 90.4 |
| Glucose 6 phosphate dehydrogenase deficiency syndrome | 80 | 65 | 81.3 |
| Egg Allergy | 78 | 57 | 73.1 |
| New drug allergy | 943 | 606 | 64.2 |
| Stop order | 1116 | 21 | 1.88 |
| **Total** | 2,794,296 | 2,615,306 | 93.6 |

Alerts generated per month and percentages overridden from inpatient setting

| Months  Alerts generated  (percent overridden) | | | January | February | March | April | May | June | July | August | September | October | November | December |
| --- | --- | --- | --- | --- | --- | --- | --- | --- | --- | --- | --- | --- | --- | --- |
| Duplicate | | 2015 |  |  |  |  |  | 29,341  (94.9) | 27,882  (94.3) | 36,584  (95.3) | 32,201  (95.1) | 42,407  (95.5) | 44,465  (96.1) | 51,137  (95.4) |
|  |  | 2016 | 51,955  (96.1) | 51,285  (96.1) | 57,444  (94.4) | 57,621  (95.8) | 61,688  (94.1) | 45,558  (96.8) | 42,196  (94.3) | 61,982  (91.3) | 56,436  (95.9) | 91,509  (93.6) | 91,593  (97.1) | 96,889  (92.3) |
|  |  | 2017 | 96,558  (94.7) | 89,874  (93.4) | 100.637 (92.6) | 91,871  (92.1) | 83,789  (96.0) | 89,381  (88.5) | 104,965 (92.0) | 100,011 (88.9) | 89,061  (85.7) | 99,711  (91.3) | 98,151  (86) | 96,194  (87.6) |
| Dose Range | | 2015 |  |  |  |  |  | 8,243  (96.5) | 7,620  (95.9) | 8,016  (98) | 6,345  (97.3) | 8,579  (96.7) | 8,126  (97.4) | 9,297  (97.8) |
|  |  | 2016 | 9,658  (97.8) | 8,651  (98.3) | 9,422  (97.1) | 8,792  (97.4) | 8,336  (98.1) | 5,940  (98.4) | 4,964  (98.0) | 7,344  (97.6) | 6,418  (97.8) | 8,628  (97.8) | 8,403  (97.9) | 9,061  (97.7) |
|  |  | 2017 | 9,229  (97.6) | 8,828  (98.2) | 12,485  (97.9) | 12,540  (98.4) | 12,539  (98.3) | 9261  (98.7) | 11,438  (98.3) | 10,764  (98.1) | 9,951  (97.9) | 11,252  (98) | 11,617  (98.7) | 11,253  (98.6) |
| Drug Interaction | | 2015 |  |  |  |  |  | 2,904  (98.3) | 2,548  (98.4) | 2,780  (97.4) | 2,911  (98.7) | 3,472  (97.8) | 3,438  (98.2) | 5,072  (97.2) |
|  |  | 2016 | 5,095  (98.6) | 4,006  (98.9) | 4,430  (98.9) | 4,058  (98.5) | 4,552  (98.9) | 3,691  (99.3) | 2,878  (97.7) | 4,195  (97.6) | 3,038  (98.5) | 4,605  (98.5) | 5,450  (98.9) | 5,547  (98.9) |
|  |  | 2017 | 6,308  (99.5) | 5,877  (99.5) | 17,964  (93.2) | 18,123  (96.3) | 18,880  (97.1) | 14,080  (95.1) | 17,603  (97.6) | 15,670  (96.9) | 14,349  (97.3) | 18,237  (96.8) | 19,138  (97.9) | 17,914  (97.6) |
| Drug allergy | | 2015 |  |  |  |  |  | 239  (90.8) | 115  (94.8) | 274  (93.4) | 178  (89.9) | 358  (90.8) | 211  (93.8) | 233  (91.4) |
|  |  | 2016 | 562  (96.4) | 313  (94.6) | 369  (94.3) | 146  (95.9) | 413  (92.3) | 125  (92.8) | 363  (92.6) | 326  (94.8) | 275  (90.6) | 270  (93) | 476  (94.3) | 311  (95.5) |
|  |  | 2017 | 301  (90.4) | 472  (86.4) | 415  (86.5) | 272  (92.6) | 485  (93.8) | 333  (89.2) | 224  (87.9) | 237  (84.4) | 232  (92.7) | 210  (88.6) | 268  (86.2) | 222  (94.1) |
| New drug allergy | | 2015 |  |  |  |  |  | 27  (74.1) | 15  (60.0) | 32  (65.6) | 33  (75.8) | 49  (59.2) | 34  (64.7) | 11  (72.7) |
|  |  | 2016 | 25  (84.0) | 26  (65.4) | 40  (55.0) | 39  (46.2) | 42  (97.6) | 20  (45.0) | 16  (100.0) | 25  (84.0) | 26  (96.2) | 41  (53.7) | 15  (73.3) | 43  (51.1) |
|  |  | 2017 | 27  (63.0) | 40  (33.0) | 29  (51.7) | 30  (36.6) | 40  (77.5) | 13  (69.2) | 21  (71.4) | 52  (59.6) | 23  (60.9) | 40  (52.5) | 48  (64.6) | 21  (90.5) |
| Egg allergy | | 2015 |  |  |  |  |  | 3  (66.7) | 1  (0) | 1  (0) | 1  (0) | 8  (75.0) | 24  (91.7) | 0  (0) |
|  |  | 2016 | 0  (0) | 0  (0) | 4  (100.0) | 0  (0) | 3  (66.7) | 0  (0) | 2  (100.0) | 0  (0) | 3  (100.0) | 3  (100.0) | 3  (66.7) | 1  (0) |
|  |  | 2017 | 0  (0) | 4  (50.0) | 2  (100.0) | 4  (100.0) | 0  (0) | 0  (0) | 0  (0) | 1  (100.0) | 2  (50.0) | 4  (25.0) | 3  (0) | 1  (0) |
| Glucose-6 phosphate dehydrogenase deficiency syndrome | | 2015 |  |  |  |  |  | 0  (0) | 0  (0) | 0  (0) | 0  (0) | 0  (0) | 4  (50.0) | 2  (100.0) |
|  |  | 2016 | 9  (88.9) | 6  (33.3) | 5  (100.0) | 4  (75.0) | 9  (66.7) | 7  (85.7) | 5  (100.0) | 5  (100.0) | 3  (100.0) | 5  (100.0) | 3  (33.3) | 4  (100.0) |
|  |  | 2017 | 0  (0) | 0  (0) | 0  (0) | 0  (0) | 1  (0) | 1  (100.0) | 1  (100.0) | 1  (100.0) | 1  (100.0) | 2  (50.0) | 1  (100.0) | 4  (75.0) |
| Height weight | 2015 | |  |  |  |  |  | 2,499  (83.7) | 2,225  (85.1) | 2,302  (88.9) | 1,699  (91.4) | 1,784  (90.3) | 1,855  (88.5) | 1,711  (88.7) |
|  | 2016 | | 1,777  (89.1) | 1,760  (85.4) | 2,112  (90.2) | 1,630  (90.6) | 1,802  (90.1) | 1,292  (91.5) | 895  (89.6) | 1,254  (90.6) | 1,428  (92.2) | 1,322  (90.3) | 1,216  (90.4) | 1,300  (91.3) |
|  | 2017 | | 1,207  (90.9) | 1,257  (93.6) | 1,154  (93.2) | 1,483  (93.2) | 1,707  (89.9) | 1,388  (91.0) | 1,374  (92.8) | 1,249 (92.8) | 1,157  (94.4) | 1,196  (93.4) | 1,236  (92.9) | 1,096  (90.6) |
| Vincristine | 2015 | |  |  |  |  |  | 0  (0) | 1  (100.0) | 0  (0) | 0  (0) | 0  (0) | 0  (0) | 1  (100.0) |
|  | 2016 | | 0  (0) | 1  (100.0) | 0  (0) | 1  (100.0) | 0  (0) | 0  (0) | 2  (50.0) | 1  (100.0) | 1  (100.0) | 0  (0) | 0  (0) | 1  (100.0) |
|  | 2017 | | 0  (0) | 0  (0) | 2  (100) | 1  (100) | 0  (0) | 0  (0) | 1  (100.0) | 2  (100.0) | 2  (100.0) | 0  (0) | 0  (0) | 0  (0) |
| Switch from injectable to oral dosage form | 2015 | |  |  |  |  |  | 1,521  (98.6) | 1,135  (98.1) | 1,629  (98.5) | 1,759  (98.4) | 2,065  (93.2) | 2,172  (97.7) | 2,727  (97.7) |
|  | 2016 | | 2,757  (97.8) | 2,684  (99.3) | 2910  (98.7) | 1,969  (98.5) | 0  (0) | 0  (0) | 0  (0) | 0  (0) | 0  (0) | 0  (0) | 0  (0) | 0  (0) |
|  | 2017 | | 0  (0) | 0  (0) | 0  (0) | 0  (0) | 0  (0) | 0  (0) | 0  (0) | 0  (0) | 0  (0) | 0  (0) | 0  (0) | 0  (0) |
| Stop order | 2015 | |  |  |  |  |  | 0  (0) | 0  (0) | 0  (0) | 0  (0) | 0  (0) | 0  (0) | 0  (0) |
|  | 2016 | | 0  (0) | 0  (0) | 0  (0) | 0  (0) | 126  (0) | 51  (0) | 47  (4.3) | 61  (13.1) | 70  (0) | 92  (0) | 89  (2.2) | 126  (0) |
|  | 2017 | | 74  (1.4) | 38  (0) | 29  (0) | 51  (0) | 52  (0) | 7  (0) | 29  (10.3) | 25  (0) | 81  (4.9) | 19  (0) | 41  (0) | 8  (0) |

Appendix G: Alerts generated and overridden from outpatient setting.

| **Alert type** | **Alerts generated** | **Alerts overridden** | |
| --- | --- | --- | --- |
|  | Number of alerts | Number of alerts | (%) of this type |
| Duplicate drug | 1,379,360 | 1,350,775 | 97.9 |
| Dose range | 208,830 | 203,977 | 97.6 |
| Drug interaction | 59,996 | 58,374 | 97.3 |
| Drug allergy | 2,959 | 2,649 | 89.5 |
| Documentation of height and weight | 610 | 454 | 74.4 |
| Egg Allergy | 148 | 88 | 59.5 |
| G6PD-syndrome | 2 | 1 | 50.0 |
| Switch medication from injectable dosage form to oral | 17 | 7 | 41.2 |
| New drug allergy | 512 | 112 | 21.9 |
| Stop order | 0 | 0 | 0.0 |
| Vincristine | 0 | 0 | 0.0 |
| **Total** | 1,652,434 | 1,616,437 | 97.8 |

Alerts generated per month and percentages overridden from outpatient setting

| Months  Alerts generated  (percent overridden) | | | January | February | March | April | May | June | July | August | September | October | November | December |
| --- | --- | --- | --- | --- | --- | --- | --- | --- | --- | --- | --- | --- | --- | --- |
| Duplicate | | 2015 |  |  |  |  |  | 8,245  (87.5) | 7,062  (88.0) | 18,233  (95.6) | 21,625  (96.8) | 36,630  (97.2) | 40,647  (98.3) | 42,082  (98.2) |
|  |  | 2016 | 39,129  (98.1) | 41,889  (98.5) | 42,315  (98.5) | 45,573  (98.6) | 59,103  (98.6) | 33,978  (98.3) | 28,408  (98.3) | 46,445  (98.3) | 36,334  (98.1) | 63,638  (98.3) | 58,916  (98.7) | 53,561  (98.7) |
|  |  | 2017 | 58,569  (98.7) | 52,213  (98.4) | 62,081  (95.3) | 60,952  (97.1) | 72,559  (98.2) | 26,826  (98.1) | 47,429  (98.0) | 40,144  (98.2) | 45,833  (98.1) | 67,138  (98.2) | 63,213  (98.3) | 58,590  (98.3) |
| Dose Range | | 2015 |  |  |  |  |  | 5,094  (96.7) | 2,513  (94.3) | 4,931  (96.6) | 4,709  (96.5) | 6,910  (97.4) | 7,290  (98.3) | 7,132  (97.9) |
|  |  | 2016 | 6,317  (97.5) | 6,393  (98.4) | 6,774  (97.4) | 6,770  (98.0) | 8,240  (97.6) | 4,599  (97.8) | 4,132  (98.3) | 6,233  (98.0) | 4,284  (98.5) | 8,048  (97.4) | 8,019  (97.7) | 6,944  (97.6) |
|  |  | 2017 | 7,848  (97.6) | 7,389  (97.8) | 8,053  (97.8) | 8,029  (96.8) | 9,659  (98.5) | 3,656  (97.9) | 8,208  (98.3) | 6,571  (98.3) | 6,807  (97.8) | 10,224  (98) | 8,751  (97.5) | 8,303  (97.4) |
| Drug interaction | | 2015 |  |  |  |  |  | 103  (79.6) | 40  (95.0) | 108  (93.5) | 161  (96.3) | 175  (96.6) | 232  (94.8) | 244  (98.8) |
|  |  | 2016 | 239  (94.6) | 192  (99.0) | 169  (92.9) | 233  (99.6) | 323  (98.8) | 168  (97.6) | 84  (100.0) | 118  (98.3) | 148  (99.3) | 366  (95.9) | 411  (99.0) | 288  (99.3) |
|  |  | 2017 | 354  (96.9) | 299  (99.3) | 6,038  (93.7) | 6,679  (97.4) | 7,409  (98.4) | 3,092  (96.0) | 4,200  (96.2) | 4,076  (97.2) | 4,574  (97.8) | 6,681  (98.4) | 6,209  (98.5) | 6,583  (98.2) |
| Drug allergy | | 2015 |  |  |  |  |  | 28  (71.4) | 25  (76.0) | 57  (82.5) | 39  (87.2) | 64  (82.8) | 83  (89.2) | 87  (90.8) |
|  |  | 2016 | 96  (88) | 78  (93.6) | 135  (90.4) | 91  (93.6) | 152  (90.4) | 71  (81.3) | 74  (90.8) | 89  (93.0) | 86  (100) | 141  (91.0) | 149  (87.2) | 126  (87.2) |
|  |  | 2017 | 131  (87.0) | 101  (96.0) | 159  (80.5) | 112  (97.3) | 135  (91.9) | 43  (93.0) | 93  (87.1) | 77  (88.3) | 57  (96.5) | 118  (95.8) | 137  (91.2) | 125  (86.4) |
| New drug allergy | | 2015 |  |  |  |  |  | 15  (86.7) | 7  (57.1) | 22  (90.1) | 16  (0.25) | 33  (33.3) | 24  (0.38) | 27  (25.9) |
|  |  | 2016 | 30  (23.3) | 13  (7.7) | 17  (17.7) | 25  (12.0) | 32  (9.4) | 7  (0.0) | 20  (15.0) | 21  (19.0) | 3  (33.3) | 3  (66.7) | 12  (83.3) | 9  (11.1) |
|  |  | 2017 | 15  (26.7) | 19  (15.8) | 22  (13.6) | 18  (16.7) | 14  (21.4) | 4  (0.0) | 6  (0.0) | 14  (0.0) | 3  (0.0) | 20  (0.0) | 20  (45.0) | 21  (38.1) |
| Egg Allergy | | 2015 |  |  |  |  |  | 2  (100.0) | 0  (0.0) | 0  (0.0) | 4  (25.0) | 14  (85.7) | 7  (85.7) | 10  (50.0) |
|  |  | 2016 | 1  (100.0) | 1  (100.0) | 4  (75.0) | 10  (50.0) | 5  (80.0) | 0  (0.0) | 0  (0.0) | 0  (0.0) | 4  (75.0) | 14  (64.3) | 10  (70.0) | 2  (50.0) |
|  |  | 2017 | 1  (100.0) | 0  (0.0) | 3  (66.7) | 0  (0.0) | 2  (50.0) | 0  (0.0) | 6  (83.3) | 2  (50.0) | 4  (75.0) | 23  (26.1) | 17  (47.1) | 2  (50.0) |
| Glucose-6 phosphate dehydrogenase deficiency syndrome | 2015 | |  |  |  |  |  | 0  (0.0) | 0  (0.0) | 0  (0.0) | 0  (0.0) | 0  (0.0) | 0  (0.0) | 0  (0.0) |
|  | 2016 | | 0  (0.0) | 0  (0.0) | 0  (0.0) | 0  (0.0) | 0  (0.0) | 0  (0.0) | 0  (0.0) | 0  (0.0) | 0  (0.0) | 1  (100.0) | 0  (0.0) | 0  (0.0) |
|  | 2017 | | 0  (0.0) | 0  (0.0) | 0  (0.0) | 0  (0.0) | 0  (0.0) | 0  (0.0) | 0  (0.0) | 0  (0.0) | 0  (0.0) | 0  (0.0) | 0  (0.0) | 0  (0.0) |
| Height weight allergy | 2015 | |  |  |  |  |  | 22  (90.1) | 10  (90.0) | 1  (100.0) | 6  (50.0) | 13  (92.3) | 11  (91.0) | 10  (20.0) |
|  | 2016 | | 20  (80.0) | 13  (100.0) | 5  (40.0) | 18  (61.1) | 14  (92.9) | 41  (70.7) | 18  (61.1) | 31  (90.3) | 14  (64.3) | 23  (91.3) | 20  (50.0) | 25  (64.0) |
|  | 2017 | | 52  (69.2) | 31  (71.0) | 21  (81.0) | 7  (71.4) | 23  (73.9) | 15  (80.0) | 19  (73.7) | 24  (91.7) | 10  (100.0) | 17  (82.4) | 35  (97.1) | 41  (80.5) |
| Switch from injectable form to oral | 2015 | |  |  |  |  |  | 0  (0) | 0  (0) | 2  (50.0) | 2  (0) | 0  (0) | 3  (100.0) | 4  (25.0) |
|  | 2016 | | 2  (50.0) | 1  (100.0) | 0  (0.0) | 3  (0.0) | 0  (0.0) | 0  (0.0) | 0  (0.0) | 0  (0.0) | 0  (0.0) | 0  (0.0) | 0  (0.0) | 0  (0.0) |
|  | 2017 | | 0  (0.0) | 0  (0.0) | 0  (0.0) | 0  (0.0) | 0  (0.0) | 0  (0.0) | 0  (0.0) | 0  (0.0) | 0  (0.0) | 0  (0.0) | 0  (0.0) | 0  (0.0) |

Appendix H: The rates of alert override across years of study

| Alert type | Duplicate | Dose range | Drug interaction | Drug allergy | New drug allergy | Egg allergy | Glucose-6 phosphate dehydrogenase deficiency | Height weight | Vincristine | Switch from Injectable to oral dosage form | Stop order |
| --- | --- | --- | --- | --- | --- | --- | --- | --- | --- | --- | --- |
| P value | <0.000 | <0.000 | <0.000 | <0.000 | <0.000 | <0.000 | <0.000 | <0.000 | 0.233 | Not assessed | Not assessed |

Appendix I: Classification into categories the examples of free text entries of reasons for overriding alerts

The first column is the classification of reasons for overriding into categories, examples of what was entered in the system have been documented in the third column of this table. They may include spelling mistakes, unclear abbreviations, mixtures of sentence case or upper case. They have been documented ‘as entered’ without modification to give a representation of entries.

| **Classification of what was entered** | **Number assigned** | **Examples of what was entered (as entered)** |
| --- | --- | --- |
| No Overridden Reason Entered | 1 | This is chosen from the pulldown menu and there is no text entered |
| Nonsensical characters | 2 | .X  ok  N  S  C  Gjgfh  ZZZ  CCCC  OJ |
| Special instructions (3A to 3D) | | |
| As instructed by physician or consulted other health care practitioner | 3A | -as order by consultant dr._ _ _ _ _  -AS PER CONSULTANT ER  -allowed by doctor  -DR DECISOON  -AS PER DR _ _ _ _ _ _ _ ORDER  -AS per RDU nurse  -Approved by Dr _ _ _ _ _ Clinical pharmacist  -as per Dr. _ _ _ _ _ clinical pharmacist  -AS PER CLINICAL PHARMASICT FOR LOW PEAK AND LEVEL TO GIVE STAT DOSE |
| As per protocol or policy | 3B | -As per protocol  -As per guidelines  -acc to AAP american association of peds its not contraindicated and considered safe  -has mild allergy to egg containing things acc to AAP american association of peds not contraindicated  -Other DKA protocol |
| Order modification; justification of ordering special instructions | 3C | -REODER  -Stopped medication awhile ago  -tapering dose  -NEED LOWER DOSE  -enoxaparin to discontinue  -Instructions provided to patient to start medication one after other and not in combination  -patient will stop enoxaparin  -Cont on isotretinoin 30 mg PO OD  -Cont on oral D/C topical  -PATIENT WAS ON IT  -in dialysate bags only  -pre op (single dose) for pt will go to cystoscopy and he is allergic to cefuroxime  -intra op if needed  -Pt off tri-luma for 3 motnhs, requested to start medication 3 weeks of eldoquine 4%  -REFILL ONLY  -FOR CT SCAN  -daily dose will be 1.9 g which is less than 2 g  -HAS KIDNEY INJURY CANT AFFORD METFORMIN OR GLICALZID  -patient already on this medication with normal urea and creatinine  -patient on this dose since admission, urea and creatinine normal  -target dose 15 mg /kg /day q8hr |
| Controlled clinical trial | 3D | -Controlled Clinical Trial  -Clinical Trial |
| Indication | 4 | -INFETILITY  -INFERTILOITY  -Infection  -PROSTATE CANCER  -THYROID CANCER  -PCO |
| Laboratory investigations or clinical findings | 5 | -PAtientvon current medication and U&E normal  -Patient weight is 15 kg  -this patient has only 1 kidney with a good function as per last labs.  -pt post liver tranplant with elevated liver enzymes  -WEIGHT 34 KG  -WEIGHT IS 25.7  -low potassium 2.9  -high glucose  -low potassium  -aptt is 38  -Normal Urea and Creatinine  -Patient with normal Cr Clearance  -low potassium and NGT suctioning  -current glucoheck is 7.9  -K level 3.2 with frequent PVCs |
| Patient centered factors (6A up to 6C) | | |
| Patient special circumstances | 6A | -outside riyadh  -mother stopped straterra more than a year  -finished, lost  -Child need syrup  -To avoid gastric upset  -maintenance done and she in on peritoneal dialysis  -pt is struggeling with refills that are not syncronised. thus pt was given all prescriptions today  -Patient is on these medications from his primary physician.  -vomitnig  -induction of sputum |
| Patient for procedure or pre-operation | 6B | -PRE OP  -As prophylaxis to be givin oncall to OR  -as the patient will go for AVF procedure tomorrow  -FOR TESTING PURPOSES  -only because she is going to OR tomorrow  -GIVE 366 mg ONCE UPON INDUCTION TO OR |
| Patient post- procedure or operation | 6C | -POST OP PROTOCOL FOR SCOLIOSIS DR._ _ _ _ _ _ _ _ _ _.  -dr._ _ _ _ _ _ _ protocol post op scoliosis  -post op |
| Aware of interaction (interaction with drug or aware of drug-allergy) | 7 | -No intraction between MTX and Infliximab  -clincal judgment  -no risks  -patient brought adol (which is paracetamol ) and she said she is taking at regularly without allergy |
| Unavailability of medication in hospital, or unavailability of medication in a certain dosage form | 8 | -THERE IS NO ORAL PIPRACILLINE  -Pharmacy do not have 0.9% NaCl  -cefotaxima not available  -we need 300mg q6hr PRN, but it is not available as a syrup.  -no oral form |
| Correction (dose or route or frequency or dosage form) of previously placed order | 9 | -CONSECUTIVE DOSES  -THE ACCURATE DOSE IS 4.5 MG / KG / DOSE  -accurate dose 360 mg  -daily dose will be 1.9 g which is less than 2 g  -Other (change Injection to tablet).  -Stopping IV and starting Oral  -i calculated highest dose according to her weight is 11 u  -15mg /kg  -Bolus  -Meningtic dose 300 mg/kg/day = 100 mg/kg/dose Q8hrs |
| Incomplete information | 10 | -DILATING DROPS  -NEED IT  -agrree to  -accepted  -?meropenem  -OK  -change  -Renal Adjustment |
| This new order is duplicate of an older no longer valid order | 11 | -We dicontinue insulin  -will cancel the older order  -Buscban is ONLY PRN  -LAST DOSE IS MIDNIGHT TODAY 21/9/2015  -THe previous order is discontinued |
| Error in alert display | 12 | -No mention which drug  -No mention which drug!!!!  -The eGFR is about 60 ml/min not < 30 as calculated by the system  -within the allowed does, window error  -No idea which medicine is being mentioned. No intimation of the name of drug. |
| Continuation of previously approved dose or combination that the patient has been taking at home or during this admission or previous admissions | 13 | -pt is recieving this at home.  -Patient on this medication from his physician in clinic  -HOME MEDICATIONS  -Patient is already on this medication for long time. He is under care of a physician already who chose to continue the medication  -Patient already on this dose  -Patient taking this combination at home  -Patient on this dose while admitted to the hospital |
| Other | 14 | -She is not on heparin  -crrt syringes  -PLEASE CHECK IF there is no interaction between those drugs. |

Appendix J: The percentage and number of alerts assessed to be displayed appropriately for each alert type in the inpatient setting.

| **Alert type** | **Number assessed** | **Assessed as appropriately displayed** | **Percentage of alerts appropriately displayed** |
| --- | --- | --- | --- |
| Egg allergy | 18 | 18 | 100.0 |
| Glucose 6 Phosphate dehydrogenase deficiency syndrome | 16 | 16 | 100.0 |
| New drug allergy | 16 | 16 | 100.0 |
| Drug allergy | 16 | 15 | 93.8 |
| Drug interaction | 15 | 13 | 86.7 |
| Dose range | 14 | 11 | 78.6 |
| Drug duplicate | 16 | 12 | 75.0 |
| Documentation of height and weight | 16 | 9 | 56.3 |
| Vincristine | 11 | 5 | 45.5 |
| Switch medication from injectable form to oral | 16 | 7 | 43.8 |
| **Total** | 154 | 122 | 79.2 |

Appendix K: The percentage and number of alerts assessed to be displayed appropriately for each alert type in the outpatient setting.

| **Alert type** | **Number of alerts assessed** | **Number of alerts assessed as appropriately displayed** | **Percentage of alerts appropriately displayed** |
| --- | --- | --- | --- |
| Dose range | 22 | 22 | 100.0 |
| Egg allergy | 22 | 22 | 100.0 |
| Switch medication from injectable form to oral | 2 | 2 | 100.0 |
| Documentation of height and weight | 22 | 17 | 81.0 |
| Drug interaction | 22 | 16 | 76.2 |
| Drug allergy | 21 | 16 | 76.2 |
| Drug duplicate | 21 | 16 | 72.7 |
| New drug allergy | 21 | 11 | 50.0 |
| **Total** | 153 | 122 | 79.7 |

Appendix L: The percentage and number of alerts overridden appropriately for each alert type in the inpatient setting.

|  | **Number of overridden alerts assessed** | **Number of alerts assessed to be overridden appropriately** | **Percentage of alerts overridden appropriately** |
| --- | --- | --- | --- |
| Egg allergy | 18 | 18 | 100 |
| Switch medication from injectable form to oral | 16 | 16 | 100 |
| Drug interaction | 15 | 15 | 100 |
| Glucose 6 Phosphate dehydrogenase deficiency syndrome. | 16 | 15 | 93.8 |
| Drug duplicate | 16 | 14 | 87.5 |
| Documentation of height and weight | 16 | 13 | 81.3 |
| Dose range | 14 | 10 | 71.4 |
| Vincristine | 11 | 7 | 63.6 |
| Drug allergy | 16 | 9 | 56.3 |
| New drug allergy | 16 | 7 | 43.8 |
| **Total** | 154 | 124 | 80.5 |

Appendix M: The percentage and number of alerts overridden appropriately for each alert type in the outpatient setting.

| **Alert type** | **Number of overridden alerts assessed** | **Number of alerts assessed as appropriately overridden** | **Percentage of alerts overridden appropriately** |
| --- | --- | --- | --- |
|  |  |  |  |
|  |  |  |  |
|  |  |  |  |
|  |  |  |  |
| Dose range | 22 | 22 | 100.0 |
| Switch medication from injectable form to oral | 2 | 2 | 100.0 |
| Egg allergy | 22 | 20 | 90.9 |
| Documentation of height and weight | 22 | 19 | 86.4 |
| Drug duplicate | 21 | 18 | 85.7 |
| Drug interaction | 22 | 16 | 72.7 |
| Drug allergy | 21 | 14 | 66.7 |
| New allergy drug | 21 | 9 | 42.9 |
| **Total** | 153 | 120 | 78.4 |

Appendix N: Meaning of interaction and allergy categories.

| Lexicomp interaction category | **Lexicomp interaction analysis** | **Description** |
| --- | --- | --- |
| A | No known interaction | Data have not demonstrated either pharmacodynamic or pharmacokinetic interactions between the specified agents. |
| B | No action needed | Data demonstrate that the specified agents may interact with each other, but there is little to no evidence of clinical concern resulting from their concomitant use. |
| C | Monitor therapy | Data demonstrate that the specified agents may interact with each other in a clinically significant manner. The benefits of concomitant use of these two medications usually outweigh the risks. An appropriate monitoring plan should be implemented to identify potential negative effects. Dosage adjustments of one or both agents may be needed in a minority of patients. |
| D | Consider therapy modification | Data demonstrate that the two medications may interact with each other in a clinically significant manner. A patient specific assessment must be conducted to determine whether the benefits of concomitant therapy outweigh the risks. Specific actions must be taken in order to realise the benefits and/or minimise the toxicity resulting from concomitant use of the agents. These actions may include aggressive monitoring, empiric dosage changes, or choosing alternative agents. |
| X | Avoid combination | Data demonstrate that the specified agents may interact with each other in a clinically significant manner. The risks associated with concomitant use of these agents usually outweigh the benefits. These agents are generally considered contraindicated. |

Appendix O: Strengthening the Reporting Observational Studies in Epidemiology Statement—Filled Checklist of items included in reporting of this study

|  | Item No | Recommendation | On Page |
| --- | --- | --- | --- |
| **Title and abstract** | 1 | (*a*) Indicate the study’s design with a commonly used term in the title or the abstract | 5 |
|  |  | (*b*) Provide in the abstract an informative and balanced summary of what was done and what was found | 4 |
| Introduction | | |  |
| Background/rationale | 2 | Explain the scientific background and rationale for the investigation being reported | 5 & 6 |
| Objectives | 3 | State specific objectives, including any prespecified hypotheses | 6 |
| Methods | | |  |
| Study design | 4 | Present key elements of study design early in the paper | 5 & 6 |
| Setting | 5 | Describe the setting, locations, and relevant dates, including periods of recruitment, exposure, follow-up, and data collection | 6 and 7 |
| Participants | 6 | (*a*) Give the eligibility criteria, and the sources and methods of selection of participants | 7 & 8 |
| Variables | 7 | Clearly define all outcomes, exposures, predictors, potential confounders, and effect modifiers. Give diagnostic criteria, if applicable | 7 |
| Data sources/ measurement | 8* | For each variable of interest, give sources of data and details of methods of assessment (measurement). Describe comparability of assessment methods if there is more than one group | 7,8,9 and appendices |
| Bias | 9 | Describe any efforts to address potential sources of bias | 8 |
| Study size | 10 | Explain how the study size was arrived at | 9 |
| Quantitative variables | 11 | Explain how quantitative variables were handled in the analyses. If applicable, describe which groupings were chosen and why | 7, 8 and 9 |
| Statistical methods | 12 | (*a*) Describe all statistical methods, including those used to control for confounding | 8 and 9 |
|  |  | (*b*) Describe any methods used to examine subgroups and interactions | 7,8,9,10,11 |
|  |  | (*c*) Explain how missing data were addressed | 13 and appendices |
|  |  | (*d*) If applicable, describe analytical methods taking account of sampling strategy | 8,9 and 13 |
|  |  | (*e*) Describe any sensitivity analyses | N/A |
| Results | | |  |
| Participants | 13* | (a) Report numbers of individuals at each stage of study—eg numbers potentially eligible, examined for eligibility, confirmed eligible, included in the study, completing follow-up, and analysed | 7,8,9,10,11 |
|  |  | (b) Give reasons for non-participation at each stage | N/A |
|  |  | (c) Consider use of a flow diagram | N/A |
| Descriptive data | 14* | (a) Give characteristics of study participants (eg demographic, clinical, social) and information on exposures and potential confounders | 13 & 14 |
|  |  | (b) Indicate number of participants with missing data for each variable of interest | N/A |
| Outcome data | 15* | Report numbers of outcome events or summary measures | 10,11,12,13 and appendices |
| Main results | 16 | (*a*) Give unadjusted estimates and, if applicable, confounder-adjusted estimates and their precision (eg, 95% confidence interval). Make clear which confounders were adjusted for and why they were included | 10 and 11 |
|  |  | (*b*) Report category boundaries when continuous variables were categorized | N/A |
|  |  | (*c*) If relevant, consider translating estimates of relative risk into absolute risk for a meaningful time period | N/A |
| Other analyses | 17 | Report other analyses done—eg analyses of subgroups and interactions, and sensitivity analyses | N/A |
| Discussion | | |  |
| Key results | 18 | Summarise key results with reference to study objectives | 10,11,12,13,14,15 |
| Limitations | 19 | Discuss limitations of the study, taking into account sources of potential bias or imprecision. Discuss both direction and magnitude of any potential bias | 14 |
| Interpretation | 20 | Give a cautious overall interpretation of results considering objectives, limitations, multiplicity of analyses, results from similar studies, and other relevant evidence | 13 & 14 |
| Generalisability | 21 | Discuss the generalisability (external validity) of the study results | 14,15 |
| Other information | | |  |
| Funding | 22 | Give the source of funding and the role of the funders for the present study and, if applicable, for the original study on which the present article is based | 19 |
